# On some new travelling wave solutions and dynamical properties of the generalized Zakharov system

**DOI:** 10.1371/journal.pone.0306319

**Published:** 2024-10-07

**Authors:** Adil Jhangeer, Kalim U. Tariq, Muhammad Nasir Ali

**Affiliations:** 1 IT4Innovations, VSB-Technical University of Ostrava, Ostrava, Poruba, Czech Republic; 2 Department of Mathematics, Namal University, Mianwali, Pakistan; 3 Department of Mathematics, Mirpur University of Science and Technology (MUST), Mirpur, AJK, Pakistan; 4 Department of Mathematics, Govt. Islamia Graduate College, Lahore, Pakistan; Federal University of Technology - Parana, BRAZIL

## Abstract

This study examines the extended version of the Zakharov system characterizing the dispersive and ion acoustic wave propagation in plasma. The genuine, non-dispersive field depicts a shift in plasma ion density from its equilibrium state, whereas the complex, dispersive field depicts the fluctuating envelope of a highly oscillatory field of electricity. The main focus of the analysis is on employing the expanded Fan sub-equation approach to achieve some novel travelling wave structures including the explicit, periodic, linked wave, and other new exact solutions are developed for different values of this parameter. Three dimensional graphs are utilised to examine the properties of the obtained solutions. Furthermore, ideas from planar dynamical theory are applied in this work to analyse the intricate behaviour of the analysed model. Sensitivity analysis, multistability, quasi-periodic and chaotic patterns, Poincaré map, and the Lyapunov characteristic exponent are used to analyse the dynamical features.

## 1 Introduction

Nonlinear partial differential equations have exceptional applications in characterizing numerous complex natural phenomena. In diverse fields of contemporary research including engineering, fluid dynamics, geochemistry, biology, elastic media, nuclear physics, astrophysics, optical fibers, acoustics, cosmology, and plasma physics, such simulations are essential for instance applied magnetism and nanophysics [1], fluid mechanics and ocean engineering [2], the ion-acoustic waves in a magnetized plasma containing cold ions and hot isothermal electrons [3] and many others. Research in such areas needs to advance with a thorough understanding of the results and other features produced from the nonlinear complex models [4]. Analytical solutions are sufficient to explain the physical attributes of a natural system, and each system solution is associated with a particular process [5]. Thus, strategies for developing exact solutions are essential to comprehend numerous nonlinear phenomenon that are getting more complex. Much research has been done to find state-of-the-art mathematical solutions for this class of issues and their analysis [6].

In recent era, scientists, technologists, mathematicians and physicists, are mostly working in this field to develop further methods, such as the auxiliary equation approach [7], the decomposition method [8], the exp-function approach [9], the sine-cosine approach [10], the Darboux transformation [11], the Hirota approach [12], the Lie group analysis [13], the similarity reduction approach [14], and the tanh approach [15–19].

In this study, travelling wave solutions of the generalised Zakharov system (GZS) are successfully constructed using the expanded Fan sub-equation approach [17]. The governing framework is written as [20]:

$$\begin{array}{c} \begin{array}{c} iY_{t}+Y_{xx}-2\sigma_{1}{\left|Y\right|}^{2}Y+2YZ=0,\\ \frac{1}{\gamma^{2}}Z_{tt}-Z_{xx}+\sigma_{2}{\left(|Y\right|}^{2}{)}_{xx}=0. \end{array}\} \end{array}$$ (1)

The actual *Z* function describes ion density fluctuations, whereas the complex *Y* function is the envelope of an oscillatory electron field; the values for *σ*_1_, *γ* and *σ*_2_ are real parameters. While *γ* that is proportional to the electron’s sound speed, implied to be the classical ZS equation when *σ*_1_ = 0 and *σ*_2_ = 1. The ZS transforms into the cubically nonlinear Schrödinger equation at sound speed *γ* → ∞, or the so-called subsonic limit.

Numerous approaches have been put forth and refined in the literature to address different kinds of ZS, for instance Melih Çınar et al. [21] applied the two different approach, namely the extended rational sine-cosine and sinh-cosh, for generalized ZS (GZS) to obtain the soliton solutions. Abbasbandy et al. [22] explored a homotopy scheme to derive approximate solutions for GZS. Additionally, Bao et al. [23] presented two efficient approximation methods time-splitting and local spectral approximations for computing the GZS and evaluated the stability and accuracy of each. The utilization of multisymplectic collocation method for approximating solutions of ZS, was presented in [24]. Furthermore, the finite difference method for dissipative ZS was analyzed in [25], where the stability of the algorithm and error bounds of approximate solutions were established. The developed solutions are newly constructed and unique because these techniques are not applied in previous literature to this model. Additionally, it is declared that the analyses performed during this work is highly valuable and of great importance in various fields of mathematical sciences, physics and many other optical transmission of data areas.

In recent years, the examination of chaotic behavior [26, 27] in systems governed by differential equations (DEs) has emerged as a prominent area of study [28]. Central to the study of DEs is the exploration of nonlinear wave behaviors and chaos theory. Understanding chaos is pivotal in contemporary times, and various methods exist for identifying chaotic patterns. This analysis emphasizes three particularly effective approaches: phase portraits, time analysis, and Lyapunov exponents [29]. Furthermore, there has been a discussion regarding the sensitivity and stability of the model across various initial conditions [30]. The techniques implemented are credible, simple, and efficient; however, they have never been applied with the governing model in the available literature. The obtained results have remarkable applications in many fields of study and in computational physics that simulate real-world situations. It is further concluded that the approaches used in this model describe the novelty of the work since these strategies have not been applied to the particular model in previous research.

This research paper is structured as follows: Section 2 presents the analytic solutions of the nonlinear systems, while Section 3 demonstrates a graphical representation of these solutions. Section 4 conducts a thorough sensitivity analysis of the initial conditions, followed by Section 5 which provides illustrations of the chaotic analysis. Subsequently, Section 6 discusses the multistability analysis of the model, and Section 7 investigates the Lyapunov characteristic exponent and Poincaré maps to ensure the manifestation of chaotic behavior. Finally, the conclusion is presented.

## 2 Solution to the generalized Zakharov system

Consider the following transformation with *γ* = *σ*_2_ = 1:

$$\begin{array}{c} Y\left(x,t\right)=\;e^{i\psi }U\left(\xi \right),\;Z\left(x,t\right)=\;V\left(\xi \right), \end{array}$$ (2)

and

$$\begin{array}{c} \psi =\alpha x+\beta t,\;\text{where}\;\;\xi =\Upsilon (x-2\alpha \;t). \end{array}$$ (3)

Using (2) and (3) into (1), we have the following equations:

$$\begin{array}{c} \Upsilon^{2}U^{\prime \prime \prime }+2UV-\left(\alpha^{2}+\beta \right)U-2\sigma_{1}U^{3}=0, \end{array}$$ (4a)

$$\begin{array}{c} \Upsilon^{2}\left(4\alpha^{2}-1\right)V^{\prime \prime }+2\Upsilon^{2}{\left(U^{2}\right)}^{\prime \prime }=0. \end{array}$$ (4b)

Integrating twice (4b) and by taking the integration constant to zero yields:

$$\begin{array}{c} V=\frac{U^{2}}{1-4\alpha^{2}},\;\alpha^{2}\neq \;\frac{1}{4}, \end{array}$$ (5)

while *c* is the constant of integration, substituting (5) into (4a) yields:

$$\begin{array}{c} \Upsilon^{2}U^{\prime \prime }+\delta_{1}U^{3}+\delta_{2}U=0, \end{array}$$ (6)

where

$$\begin{array}{ccc} \delta_{1} & = & \frac{2}{1-4\alpha^{2}}-2\sigma_{1},\\ \delta_{2} & = & 2c-\alpha^{2}-\beta . \end{array}$$ (7)

From (6), *n* = 1 is obtained with homogeneous balance. Let the solution of (6) is expressed as:

$$\begin{array}{c} U=a_{0}+a_{1}\phi \left(\xi \right), \end{array}$$ (8)

which holds the following condition:

$$\begin{array}{c} \phi^{\prime }{\left(\xi \right)}^{2}=\Omega_{0}+\Omega_{1}\phi \left(\xi \right)+\Omega_{2}\phi {\left(\xi \right)}^{2}+\Omega_{3}\phi {\left(\xi \right)}^{3}+\Omega_{4}\phi {\left(\xi \right)}^{4}. \end{array}$$ (9)

Substituting (8) along (7) in (6) and comparing the coefficients of *ϕ*^*j*^*ϕ*^(*k*)^, we have:

$$\begin{array}{ccc} \frac{1}{2}\Upsilon^{2}a_{1}\Omega_{1}+a_{0}^{3}\delta_{1}+a_{0}\delta_{2} & = & 0,\\ \Upsilon^{2}a_{1}\Omega_{2}+3a_{0}^{2}a_{1}\delta_{1}+a_{1}\delta_{2} & = & 0,\\ \frac{3}{2}\Upsilon^{2}a_{1}\Omega_{3}+3a_{0}a_{1}^{2}\delta_{1} & = & 0,\\ 2\Upsilon^{2}a_{1}\Omega_{4}+a_{1}^{3}\delta_{1} & = & 0. \end{array}$$ (10)

After solving for Ω_*i*_, (*i* = 0, 1, 2, 3, 4), we have:

$$\begin{array}{ccc} \Omega_{1} & = & -\frac{2(a_{0}^{3}\delta_{1}+a_{0}\delta_{2})}{\Upsilon^{2}a_{1}},\\ \Omega_{2} & = & -\frac{3a_{0}^{2}\delta_{1}+\delta_{2}}{\Upsilon^{2}},\\ \Omega_{3} & = & -\frac{2a_{0}a_{1}\delta_{1}}{\Upsilon^{2}},\\ \Omega_{4} & = & -\frac{a_{1}^{2}\delta_{1}}{2\Upsilon^{2}}, \end{array}$$ (11)

therefore

$$\begin{array}{c} Y\left(x,t\right)=(a_{0}+a_{1}\phi \left(\xi \right))\times e^{i\psi }, \end{array}$$ (12)

which gives

$$\begin{array}{c} Z\left(x,t\right)=\frac{Y^{2}}{1-4\alpha^{2}}, \end{array}$$ (13)

where

$$\begin{array}{c} a_{0}=\frac{\sqrt{\Omega_{2}(-\Upsilon^{2})-\delta_{2}}}{\sqrt{3}\sqrt{\delta_{1}}},\;a_{1}=\frac{\sqrt{2}\sqrt{-\Omega_{4}}\Upsilon }{\sqrt{\delta_{1}}}, \end{array}$$

and *ψ* = *αx* + *βt*. We have the following solutions as developed in [31]:

Case I.

If $\Omega_{0}=\Psi_{3}^{2},\Omega_{1}=2\Psi_{1}\Psi_{3},\Omega_{2}=2\Psi_{2}\Psi_{3}+\Psi_{1}^{2},\Omega_{3}=2\Psi_{1}\Psi_{2},\Omega_{4}=\Psi_{2}^{2}$, for some parameters Ψ_1_, Ψ_2_, and Ψ_3_, Some of the important optical solitons solutions $Y_{\eta }^{I},\left(\eta =1,2,\ldots ,24\right)$ of (1) are listed below.

Type I: When $\Psi_{1}^{2}-4\Psi_{2}\Psi_{3}>0$, Ψ_1_Ψ_2_ ≠ 0, Ψ_2_Ψ_3_ ≠ 0, we have the following dark optical soliton and bright-dark optical soliton:

$$\begin{array}{ccc} Y_{1}^{I}\left(x,t\right) & = & [\frac{\sqrt{\left(2\Psi_{2}\Psi_{3}+\Psi_{1}^{2}\right)(-\Upsilon^{2})-\delta_{2}}}{\sqrt{3}\sqrt{\delta_{1}}}+\frac{\sqrt{2}\sqrt{-\Psi_{2}^{2}}\Upsilon }{\sqrt{\delta_{1}}}\\ & & \times (\frac{\sqrt{\Psi_{1}^{2}-4\Psi_{2}\Psi_{3}}\;\text{tanh}\;(\frac{1}{2}\xi \sqrt{\Psi_{1}^{2}-4\Psi_{2}\Psi_{3}})+\Psi_{1}}{2\Psi_{2}})]\times e^{i\psi }. \end{array}$$

$$\begin{array}{lll} Y_{5}^{I}\left(x,t\right) & = & [\frac{\sqrt{\left(2\Psi_{2}\Psi_{3}+\Psi_{1}^{2}\right)\left(-\Upsilon^{2}\right)-\delta_{2}}}{\sqrt{3}\sqrt{\delta_{1}}}+\frac{\sqrt{2}\sqrt{-\Psi_{2}^{2}}\Upsilon }{\sqrt{\delta_{1}}}\\ & & \times (\sqrt{\Psi_{1}^{2}-4\Psi_{2}\Psi_{3}}(\text{tanh}\left(\frac{1}{4}\xi \sqrt{\Psi_{1}^{2}-4\Psi_{2}\Psi_{3}}\right)))\\ & & +\text{coth}\left(\frac{1}{4}\xi \sqrt{\Psi_{1}^{2}-4\Psi_{2}\Psi_{3}}\right)+\Psi_{1})]\times e^{i\psi }. \end{array}$$

Type II: When $\Psi_{1}^{2}-4\Psi_{2}\Psi_{3}<0$, Ψ_1_Ψ_2_ ≠ 0, Ψ_2_Ψ_3_ ≠ 0, we have the following bright optical soliton and singular optical soliton.

$$\begin{array}{ccc} Y_{13}^{II}\left(x,t\right) & = & [\frac{\sqrt{\left(2\Psi_{2}\Psi_{3}+\Psi_{1}^{2}\right)(-\Upsilon^{2})-\delta_{2}}}{\sqrt{3}\sqrt{\delta_{1}}}+\frac{\sqrt{2}\sqrt{-\Psi_{2}^{2}}\Upsilon }{\sqrt{\delta_{1}}}\\ & & \times (\frac{\sqrt{4\Psi_{2}\Psi_{3}-\Psi_{1}^{2}}\;\text{tan}\;(\frac{1}{2}\xi \sqrt{4\Psi_{2}\Psi_{3}-\Psi_{1}^{2}})-\Psi_{1}}{2\Psi_{2}})]\times e^{i\psi }. \end{array}$$

$$\begin{array}{ccc} Y_{24}^{II}\left(x,t\right) & = & [\frac{\sqrt{\left(2\Psi_{2}\Psi_{3}+\Psi_{1}^{2}\right)(-\Upsilon^{2})-\delta_{2}}}{\sqrt{3}\sqrt{\delta_{1}}}+\frac{\sqrt{2}\sqrt{-\Psi_{2}^{2}}\Upsilon }{\sqrt{\delta_{1}}}\\ & & \times (4\Psi_{3}\;\text{sin}\;(\frac{1}{4}\xi \sqrt{4\Psi_{2}\Psi_{3}-\Psi_{1}^{2}})\;\text{cos}\;(\frac{1}{4}\xi \sqrt{4\Psi_{2}\Psi_{3}-\Psi_{1}^{2}}))\\ & & \left[2\sqrt{4\Psi_{2}\Psi_{3}-\Psi_{1}^{2}}\;{\text{cos}}^{2}\;(\frac{1}{4}\xi \sqrt{4\Psi_{2}\Psi_{3}-\Psi_{1}^{2}})-2\Psi_{1}\;\text{sin}\;(\frac{1}{4}\xi \sqrt{4\Psi_{2}\Psi_{3}-\Psi_{1}^{2}}\right)\\ & & \text{cos}\;(\frac{1}{4}\xi \sqrt{4\Psi_{2}\Psi_{3}-\Psi_{1}^{2}})-\sqrt{4\Psi_{2}\Psi_{3}-\Psi_{1}^{2}}{]}^{-1}]\times e^{i\psi }. \end{array}$$

Case II.

If $\Omega_{0}=\Psi_{3}^{2},\Omega_{1}=2\Psi_{1}\Psi_{3},\Omega_{2}=0,\Omega_{3}=2\Psi_{1}\Psi_{2},\Omega_{4}=\Psi_{2}^{2}$, *Y* is one of the $Y_{\eta }^{II},\left(\eta =1,2,\ldots ,12\right)$. In this case, we get the following soliton solutions and bright-dark optical soliton solutions where Υ_1_, Υ_2_ are any arbitrary constants:

$$\begin{array}{ccc} Y_{1}^{II}\left(x,t\right) & = & [\frac{\sqrt{\left(2\Psi_{2}\Psi_{3}+\Psi_{1}^{2}\right)(-\Upsilon^{2})-\delta_{2}}}{\sqrt{3}\sqrt{\delta_{1}}}+\frac{\sqrt{2}\sqrt{-\Psi_{2}^{2}}\Upsilon }{\sqrt{\delta_{1}}}\\ & & \times (\frac{\sqrt{-6\Psi_{2}\Psi_{3}}\;\text{tanh}\;(\frac{1}{2}\xi \sqrt{-6\Psi_{2}\Psi_{3}})+\sqrt{-2\Psi_{2}\Psi_{3}}}{2\Psi_{2}})]\times e^{i\psi }. \end{array}$$

$$\begin{array}{ccc} Y_{6}^{II}\left(x,t\right) & = & [\frac{\sqrt{\left(2\Psi_{2}\Psi_{3}+\Psi_{1}^{2}\right)(-\Upsilon^{2})-\delta_{2}}}{\sqrt{3}\sqrt{\delta_{1}}}+\frac{\sqrt{2}\sqrt{-\Psi_{2}^{2}}\Upsilon }{\sqrt{\delta_{1}}}\\ & & \times (\frac{\sqrt{(\Upsilon_{1}^{2}+\Upsilon_{2}^{2})\left(-6\Psi_{2}\Psi_{3}\right)}-\Upsilon_{1}\sqrt{-6\Psi_{2}\Psi_{3}}\;\text{cosh}\;(\xi \sqrt{-6\Psi_{2}\Psi_{3}})}{\Upsilon_{2}+\Upsilon_{1}\;\text{sinh}\;(\xi \sqrt{-6\Psi_{2}\Psi_{3}})}\\ & & +2\sqrt{-2\Psi_{2}\Psi_{3}})]\times e^{i\psi }. \end{array}$$

$$\begin{array}{ccc} Y_{12}^{II}\left(x,t\right) & = & [\frac{\sqrt{\left(2\Psi_{2}\Psi_{3}+\Psi_{1}^{2}\right)(-\Upsilon^{2})-\delta_{2}}}{\sqrt{3}\sqrt{\delta_{1}}}+\frac{\sqrt{2}\sqrt{-\Psi_{2}^{2}}\Upsilon }{\sqrt{\delta_{1}}}\\ & & \times (4\Psi_{3}\;\text{sinh}\;(\frac{1}{4}\xi \sqrt{-6\Psi_{2}\Psi_{3}})\;\text{cosh}\;(\frac{1}{4}\xi \sqrt{-6\Psi_{2}\Psi_{3}}))\\ & & [2\sqrt{-6\Psi_{2}\Psi_{3}}\;{\text{cosh}}^{2}\;(\frac{1}{4}\xi \sqrt{-6\Psi_{2}\Psi_{3}})-\sqrt{-6\Psi_{2}\Psi_{3}}\\ & & +2\sqrt{-2\Psi_{2}\Psi_{3}}\;\text{sinh}\;(\frac{1}{4}\xi \sqrt{-6\Psi_{2}\Psi_{3}})\;\text{cosh}\;(\frac{1}{4}\xi \sqrt{-6\Psi_{2}\Psi_{3}}){]}^{-1}]\times e^{i\psi }. \end{array}$$

Case III.

If Ω_0_ = Ω_1_ = 0, solution of (1) in the form $Y_{\eta }^{III},\left(\eta =1,2,\ldots ,10\right)$ are extracted where Υ_1_, Υ_2_, Υ_3_ are constants,

Type I: When $\Omega_{2}=1,\Omega_{3}=\frac{-2\Upsilon_{3}}{\Upsilon_{1}},\Omega_{4}=\frac{\Upsilon_{3}^{2}-\Upsilon_{2}^{2}}{\Upsilon_{1}^{2}}$, we have

$$\begin{array}{ccc} Y_{1}^{III}\left(x,t\right) & = & [\frac{\sqrt{\left(2\Psi_{2}\Psi_{3}+\Psi_{1}^{2}\right)(-\Upsilon^{2})-\delta_{2}}}{\sqrt{3}\sqrt{\delta_{1}}}+\frac{\sqrt{2}\sqrt{-\Psi_{2}^{2}}\Upsilon }{\sqrt{\delta_{1}}}\times (\frac{\Upsilon_{1}\text{sech}\left(\xi \right)}{\Upsilon_{3}\text{sech}\left(\xi \right)+\Upsilon_{2}})]\times e^{i\psi }. \end{array}$$

Type II: When $\Omega_{2}=1,\Omega_{3}=\frac{-2\Upsilon_{3}}{\Upsilon_{1}},\Omega_{4}=\frac{\Upsilon_{3}^{2}+\Upsilon_{2}^{2}}{\Upsilon_{1}^{2}}$, we have

$$\begin{array}{ccc} Y_{2}^{III}\left(x,t\right) & = & [\frac{\sqrt{\left(2\Psi_{2}\Psi_{3}+\Psi_{1}^{2}\right)(-\Upsilon^{2})-\delta_{2}}}{\sqrt{3}\sqrt{\delta_{1}}}+\frac{\sqrt{2}\sqrt{-\Psi_{2}^{2}}\Upsilon }{\sqrt{\delta_{1}}}\times (\frac{\Upsilon_{1}\text{csch}\left(\xi \right)}{\Upsilon_{3}\text{csch}\left(\xi \right)+\Upsilon_{2}})]\times e^{i\psi }. \end{array}$$

Type III: When $\Omega_{2}=4,\Omega_{3}=-\frac{4(2\Upsilon_{2}+\Upsilon_{4})}{\Upsilon_{1}},\Omega_{4}=\frac{4\Upsilon_{2}^{2}+4\Upsilon_{4}\Upsilon_{2}+\Upsilon_{3}^{2}}{\Upsilon_{1}^{2}}$, we have

$$\begin{array}{ccc} Y_{3}^{III}\left(x,t\right) & = & [\frac{\sqrt{\left(2\Psi_{2}\Psi_{3}+\Psi_{1}^{2}\right)(-\Upsilon^{2})-\delta_{2}}}{\sqrt{3}\sqrt{\delta_{1}}}+\frac{\sqrt{2}\sqrt{-\Psi_{2}^{2}}\Upsilon }{\sqrt{\delta_{1}}}\times \\ & & (\frac{\Upsilon_{1}{\text{sech}}^{2}\left(\xi \right)}{\Upsilon_{3}\;\text{tanh}\left(\xi \right)+\Upsilon_{2}{\text{sech}}^{2}\left(\xi \right)+\Upsilon_{4}})]\times e^{i\psi }. \end{array}$$

Type IV: When $\Omega_{2}=4,\Omega_{3}=\frac{4(\Upsilon_{4}-2\Upsilon_{2})}{\Upsilon_{1}},\Omega_{4}=\frac{4\Upsilon_{2}^{2}-4\Upsilon_{4}\Upsilon_{2}+\Upsilon_{3}^{2}}{\Upsilon_{1}^{2}}$, we have

$$\begin{array}{ccc} Y_{4}^{III}\left(x,t\right) & = & [\frac{\sqrt{\left(2\Psi_{2}\Psi_{3}+\Psi_{1}^{2}\right)(-\Upsilon^{2})-\delta_{2}}}{\sqrt{3}\sqrt{\delta_{1}}}+\frac{\sqrt{2}\sqrt{-\Psi_{2}^{2}}\Upsilon }{\sqrt{\delta_{1}}}\times \\ & & (\frac{\Upsilon_{1}{\text{csch}}^{2}\left(\xi \right)}{\Upsilon_{3}\text{coth}\left(\xi \right)+\Upsilon_{2}{\text{csch}}^{2}\left(\xi \right)+\Upsilon_{4}})]\times e^{i\psi }. \end{array}$$

Type V: When $\Omega_{2}=-1,\Omega_{3}=\frac{2\Upsilon_{3}}{\Upsilon_{1}},\Omega_{4}=\frac{\Upsilon_{3}^{2}-\Upsilon_{2}^{2}}{\Upsilon_{1}^{2}}$, we have

$$\begin{array}{ccc} Y_{6}^{III}\left(x,t\right) & = & [\frac{\sqrt{\left(2\Psi_{2}\Psi_{3}+\Psi_{1}^{2}\right)(-\Upsilon^{2})-\delta_{2}}}{\sqrt{3}\sqrt{\delta_{1}}}+\frac{\sqrt{2}\sqrt{-\Psi_{2}^{2}}\Upsilon }{\sqrt{\delta_{1}}}\\ & & \times (-\frac{\Upsilon_{1}(\text{sinh}(\Upsilon_{1}\xi )+\text{cosh}(\Upsilon_{1}\xi ))(\text{sinh}(\Upsilon_{1}\xi )+\text{cosh}(\Upsilon_{1}\xi )+\Upsilon_{3})}{\Upsilon_{2}})]\times e^{i\psi }. \end{array}$$

Type VI: When $\Omega_{2}=4,\Omega_{3}=\frac{-2\Upsilon_{3}}{\Upsilon_{1}},\Omega_{4}=\frac{\Upsilon_{3}^{2}-\Upsilon_{2}^{2}}{\Upsilon_{1}^{2}}$, we get

$$\begin{array}{ccc} Y_{8}^{III}\left(x,t\right) & = & [\frac{\sqrt{\left(2\Psi_{2}\Psi_{3}+\Psi_{1}^{2}\right)(-\Upsilon^{2})-\delta_{2}}}{\sqrt{3}\sqrt{\delta_{1}}}+\frac{\sqrt{2}\sqrt{-\Psi_{2}^{2}}\Upsilon }{\sqrt{\delta_{1}}}\times (\frac{\Upsilon_{1}\text{csc}\left(\xi \right)}{\Upsilon_{3}\text{csc}\left(\xi \right)+\Upsilon_{2}})]\times e^{i\psi }. \end{array}$$

Type VII: When $\Omega_{2}=-4,\Omega_{3}=\frac{4(2\Upsilon_{2}+\Upsilon_{4})}{\Upsilon_{1}},\Omega_{4}=-\frac{4\Upsilon_{2}^{2}+4\Upsilon_{4}\Upsilon_{2}-\Upsilon_{3}^{2}}{\Upsilon_{1}^{2}}$, we get

$$\begin{array}{ccc} Y_{9}^{III}\left(x,t\right) & = & [\frac{\sqrt{\left(2\Psi_{2}\Psi_{3}+\Psi_{1}^{2}\right)(-\Upsilon^{2})-\delta_{2}}}{\sqrt{3}\sqrt{\delta_{1}}}+\frac{\sqrt{2}\sqrt{-\Psi_{2}^{2}}\Upsilon }{\sqrt{\delta_{1}}}\\ & & \times (\frac{\Upsilon_{1}{\text{sec}}^{2}\left(\xi \right)}{\Upsilon_{3}\text{tan}\left(\xi \right)+\Upsilon_{2}{\text{sec}}^{2}\left(\xi \right)+\Upsilon_{4}})]\times e^{i\psi }. \end{array}$$

Case IV.

If Ω_1_ = Ω_3_ = 0, solution of (1) in the form $Y_{\eta }^{IV},\left(\eta =1,2,\ldots ,16\right)$ are obtained.

When Ω_0_ = 1 − *m*^2^, Ω_2_ = 2*m*^2^ − 1, Ω_4_ = −*m*^2^, we get:

$$\begin{array}{ccc} Y_{3}^{IV}\left(\xi \right) & = & (\frac{\sqrt{\Omega_{2}(-\Upsilon^{2})-\delta_{2}}}{\sqrt{3}\sqrt{\delta_{1}}}+\frac{\sqrt{2}\sqrt{-\Omega_{4}}\Upsilon }{\sqrt{\delta_{1}}}\times cn\left(\xi \right))\times e^{i\psi }. \end{array}$$

For *m* → 1, we get combined wave solutions

$$\begin{array}{ccc} Y_{3a}^{IV}\left(\xi \right) & = & (\frac{\sqrt{\Omega_{2}(-\Upsilon^{2})-\delta_{2}}}{\sqrt{3}\sqrt{\delta_{1}}}+\frac{\sqrt{2}\sqrt{-\Omega_{4}}\Upsilon }{\sqrt{\delta_{1}}}\times \text{sec}\;h\left(\xi \right))\times e^{i\psi }, \end{array}$$

For *m* → 0, we have periodic singular solutions

$$\begin{array}{ccc} Y_{3b}^{IV}\left(\xi \right) & = & (\frac{\sqrt{\Omega_{2}(-\Upsilon^{2})-\delta_{2}}}{\sqrt{3}\sqrt{\delta_{1}}}+\frac{\sqrt{2}\sqrt{-\Omega_{4}}\Upsilon }{\sqrt{\delta_{1}}}\times \text{cos}\left(\xi \right))\times e^{i\psi }. \end{array}$$

Similarly, for $\Omega_{0}=\frac{1}{4},\Omega_{2}=\frac{1-2m^{2}}{2},\Omega_{4}=\frac{1}{4}$, we get the following solutions of (1)

$$\begin{array}{ccc} Y_{13}^{IV}\left(\xi \right) & = & (\frac{\sqrt{\Omega_{2}(-\Upsilon^{2})-\delta_{2}}}{\sqrt{3}\sqrt{\delta_{1}}}+\frac{\sqrt{2}\sqrt{-\Omega_{4}}\Upsilon }{\sqrt{\delta_{1}}}\times \left(ns\xi \pm cs\xi \right))\times e^{i\psi }. \end{array}$$

For *m* → 1, we get combined wave solutions

$$\begin{array}{ccc} Y_{13a}^{IV}\left(\xi \right) & = & (\frac{\sqrt{\Omega_{2}(-\Upsilon^{2})-\delta_{2}}}{\sqrt{3}\sqrt{\delta_{1}}}+\frac{\sqrt{2}\sqrt{-\Omega_{4}}\Upsilon }{\sqrt{\delta_{1}}}\times \left(\text{coth}\left(\xi \right)+\text{csc}\;h\left(\xi \right)\right))\times e^{i\psi }, \end{array}$$

For *m* → 0, we get the periodic singular solution

$$\begin{array}{ccc} Y_{13b}^{IV}\left(\xi \right) & = & (\frac{\sqrt{\Omega_{2}(-\Upsilon^{2})-\delta_{2}}}{\sqrt{3}\sqrt{\delta_{1}}}+\frac{\sqrt{2}\sqrt{-\Omega_{4}}\Upsilon }{\sqrt{\delta_{1}}}\times \left(\text{cot}\left(\xi \right)+\text{csc}\left(\xi \right)\right))\times e^{i\psi }. \end{array}$$

where *ξ* = Υ(*x* − 2*αt*) and *ψ* = *αx* + *βt*.

## 3 Discussions and results

Graphical representation of solitons has been illustrated in the following figures, for different values of the parameters. As a result, various traveling wave solutions are found, namely bright, dark, optical, periodic, and combined waves.

**Fig 1:**
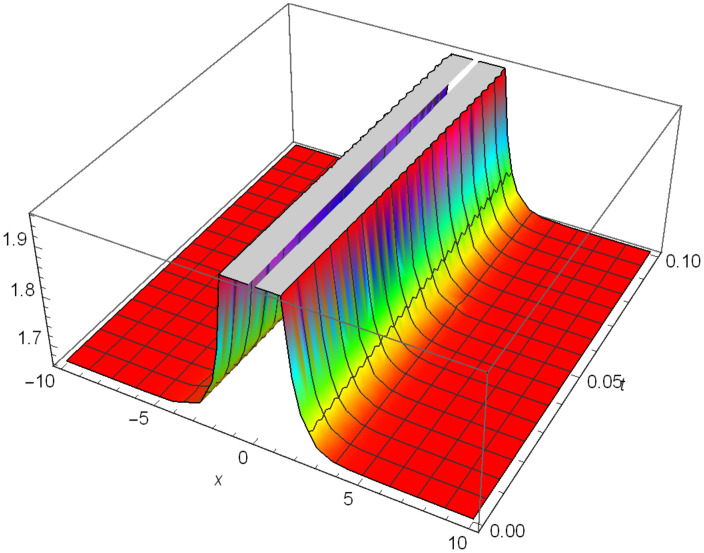
Periodic singular wave structure for the generalized Zakharov system (Case-I Type I) in 3D visualization of ${|Y\left(x,t\right)}_{5}^{I}|$: Ψ_1_ = *2*, Ψ_2_ = *0.5*, Ψ_3_ = *1*, *σ*_1_ = *1*, *σ*_2_ = *3*, *α* = *2*, *β* = *1*, Υ = -*1.25*.

**Fig 2:**
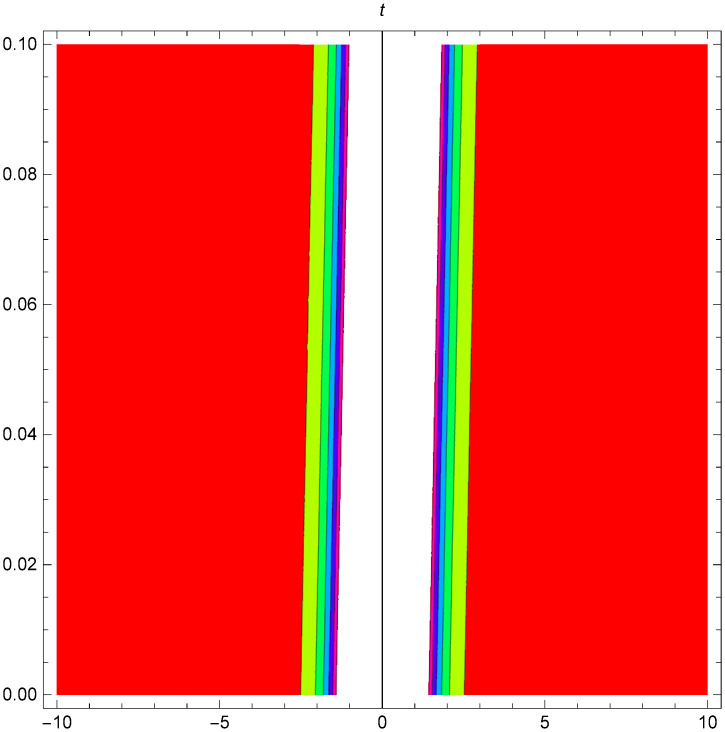
Periodic singular wave structure for the generalized Zakharov system (Case-I Type I) in Cantor shape of ${|Y\left(x,t\right)}_{5}^{I}|$: Ψ_1_ = *2*, Ψ_2_ = *0.5*, Ψ_3_ = *1*, *σ*_1_ = *1*, *σ*_2_ = *3*, *α* = *2*, *β* = *1*, Υ = -*1.25*.

**Fig 3:**
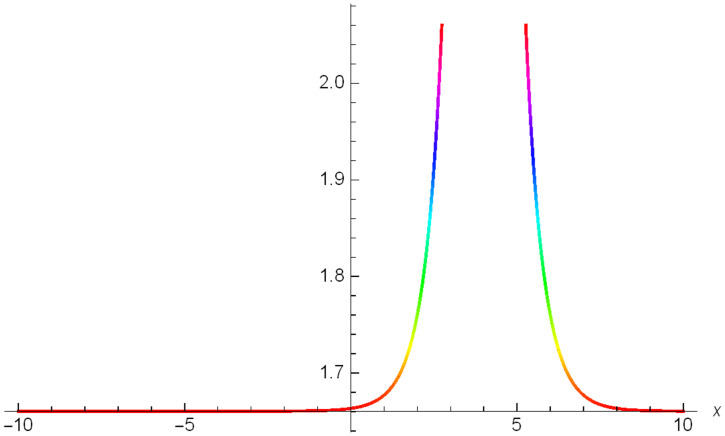
Periodic singular wave structure for the generalized Zakharov system (Case-I Type I) in 2D visualization of ${|Y\left(x,t\right)}_{5}^{I}|$: Ψ_1_ = *2*, Ψ_2_ = *0.5*, Ψ_3_ = *1*, *σ*_1_ = *1*, *σ*_2_ = *3*, *α* = *2*, *β* = *1*, Υ = -*1.25*.

Figs 1–3 illustrate a periodic singular wave structure $|Y_{5}^{I}\left(x,t\right)|$ established in Case I (Type I) for Ψ_1_ = *2*, Ψ_2_ = *0.5*, Ψ_3_ = *1*, *σ*_1_ = *1*, *σ*_2_ = *3*, *α* = *2*, *β* = *1*, Υ = -*1.25* in 3D, Cantor and 2D formats respectively.

**Fig 4:**
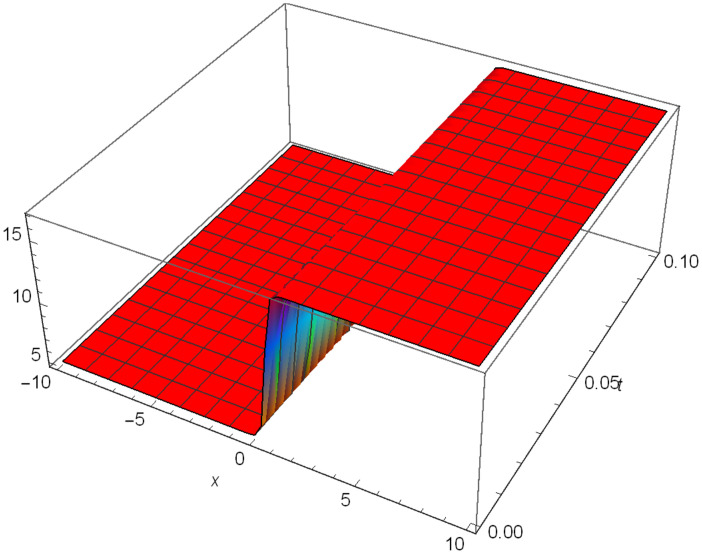
Solitary wave structure for the generalized Zakharov system (Case-I Type I) in 3D visualizations of ${|Y\left(x,t\right)}_{10}^{I}|$: Ψ_1_ = *3*, Ψ_2_ = *0.5*, Ψ_3_ = -*1*, *σ*_1_ = -*0.5*, *σ*_2_ = *3.5*, *α* = *1.25*, *β* = *2*, Υ = *1.25*.

**Fig 5:**
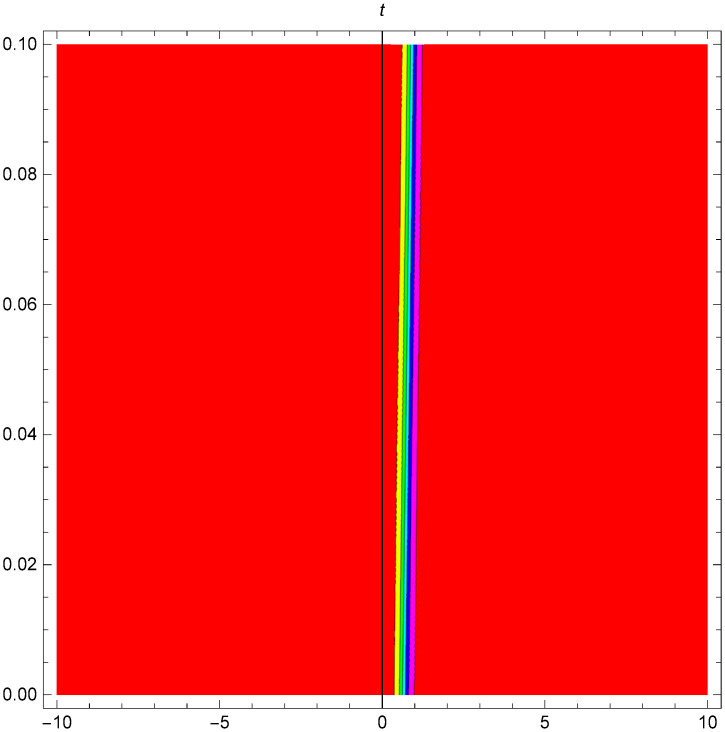
Solitary wave structure for the generalized Zakharov system (Case-I Type I) in Cantor shape of ${|Y\left(x,t\right)}_{10}^{I}|$: Ψ_1_ = *3*, Ψ_2_ = *0.5*, Ψ_3_ = -*1*, *σ*_1_ = -*0.5*, *σ*_2_ = *3.5*, *α* = *1.25*, *β* = *2*, Υ = *1.25*.

**Fig 6:**
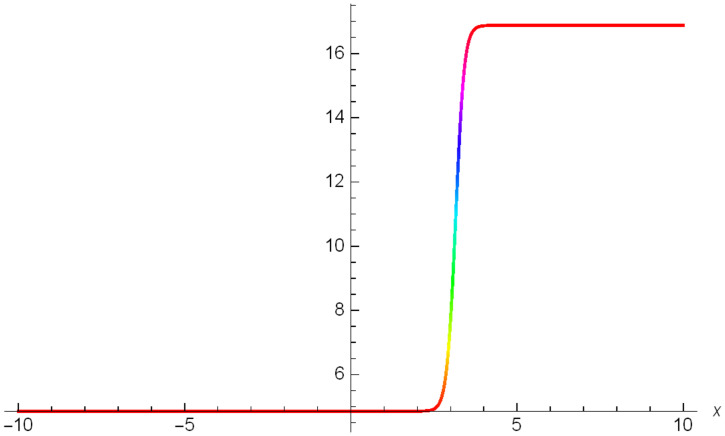
Solitary wave structure for the generalized Zakharov system (Case-I Type I) in 2D visualizations of ${|Y\left(x,t\right)}_{10}^{I}|$: Ψ_1_ = *3*, Ψ_2_ = *0.5*, Ψ_3_ = -*1*, *σ*_1_ = -*0.5*, *σ*_2_ = *3.5*, *α* = *1.25*, *β* = *2*, Υ = *1.25*.

While Figs 4–6 display a solitary wave structure $|Y_{10}^{I}\left(x,t\right)|$ established in Case I (Type I) for Ψ_1_ = *3*, Ψ_2_ = *0.5*, Ψ_3_ = -*1*, *σ*_1_ = -*0.5*, *σ*_2_ = *3.5*, *α* = *1.25*, *β* = *2*, Υ = *1.25* in 3D, Cantor and 2D formats respectively.

**Fig 7:**
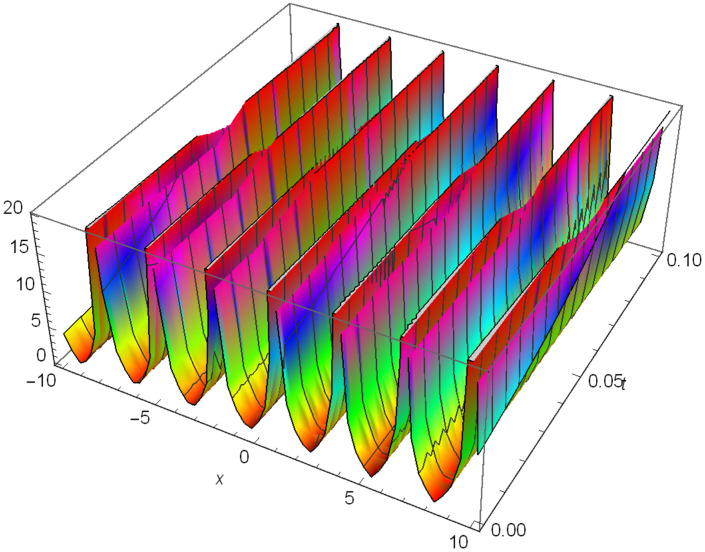
Periodic singular wave structure for the generalized Zakharov system (Case-I Type II) in 3D visualization of ${|Y\left(x,t\right)}_{24}^{I}|$: Ψ_1_ = *1*, Ψ_2_ = *1*, Ψ_3_ = *1*, *σ*_1_ = -*0.5*, *σ*_2_ = *3.5*, *α* = *1.25*, *β* = *2*, Υ = *1.25*.

**Fig 8:**
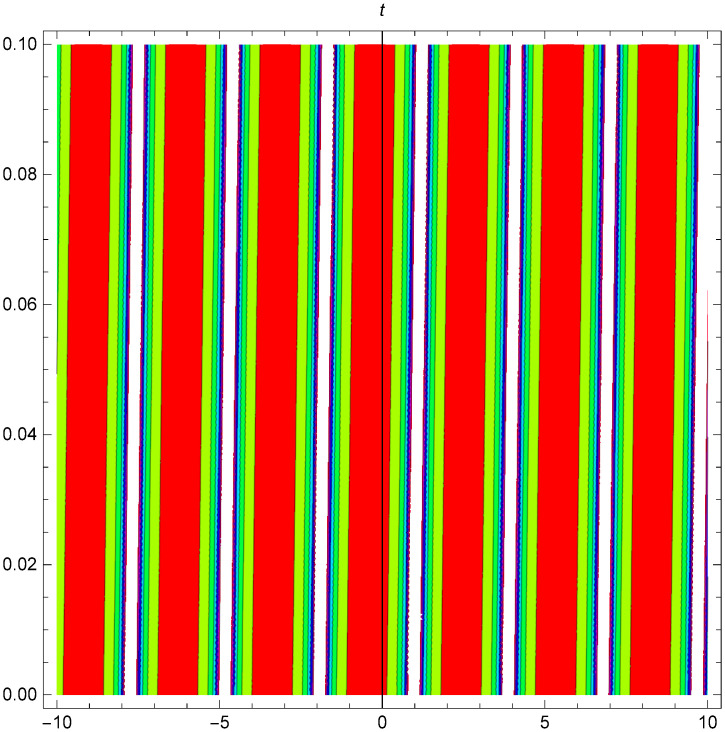
Periodic singular wave structure for the generalized Zakharov system (Case-I Type II) in Cantor shape of ${|Y\left(x,t\right)}_{24}^{I}|$: Ψ_1_ = *1*, Ψ_2_ = *1*, Ψ_3_ = *1*, *σ*_1_ = -*0.5*, *σ*_2_ = *3.5*, *α* = *1.25*, *β* = *2*, Υ = *1.25*.

**Fig 9:**
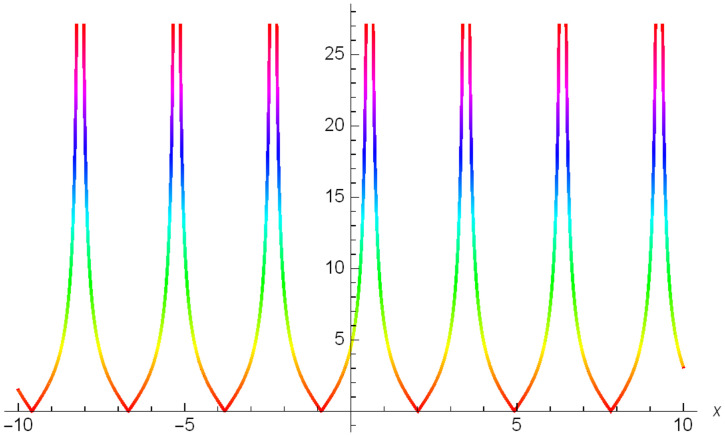
Periodic singular wave structure for the generalized Zakharov system (Case-I Type II) in 2D visualization of ${|Y\left(x,t\right)}_{24}^{I}|$: Ψ_1_ = *1*, Ψ_2_ = *1*, Ψ_3_ = *1*, *σ*_1_ = -*0.5*, *σ*_2_ = *3.5*, *α* = *1.25*, *β* = *2*, Υ = *1.25*.

Whereas Figs 7–9 demonstrate a periodic singular wave structure $|Y_{24}^{I}\left(x,t\right)|$ established in Case I (Type II) for Ψ_1_ = *1*, Ψ_2_ = *1*, Ψ_3_ = *1*, *σ*_1_ = -*0.5*, *σ*_2_ = *3.5*, *α* = *1.25*, *β* = *2*, Υ = *1.25* in 3D, Cantor and 2D formats respectively.

**Fig 10:**
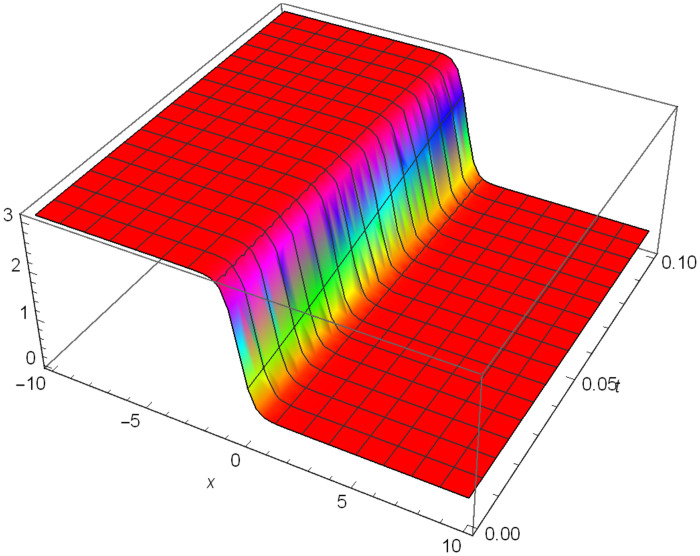
Solitary wave structure for the generalized Zakharov system (Case-II) in 3D visualization of ${|Y\left(x,t\right)}_{12}^{II}|$: Ψ_1_ = *1*, Ψ_2_ = -*1*, Ψ_3_ = *0.25*, *σ*_1_ = *2*, *σ*_2_ = *1*, *α* = *3*, *β* = *2*, Υ = *3*.

**Fig 11:**
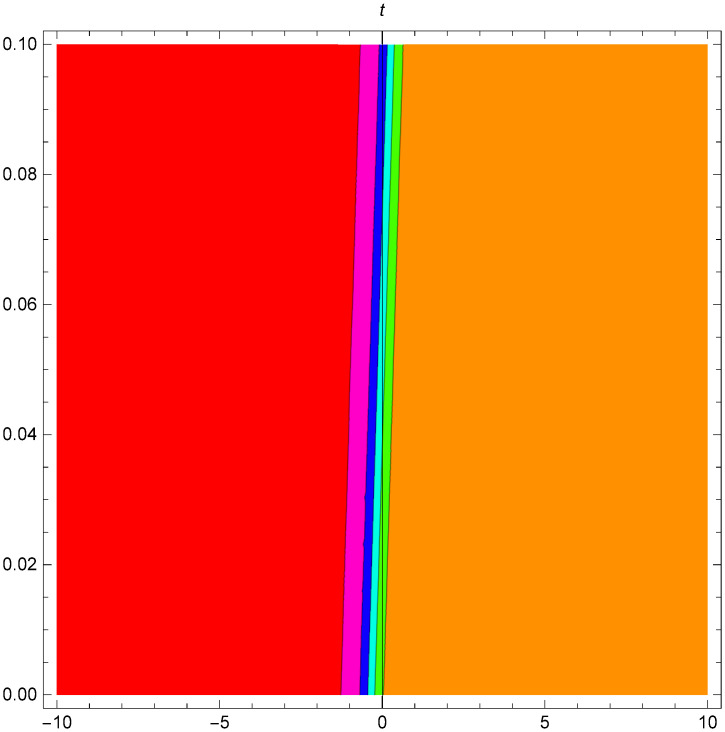
Solitary wave structure for the generalized Zakharov system (Case-II) in Canto shape of ${|Y\left(x,t\right)}_{12}^{II}|$: Ψ_1_ = *1*, Ψ_2_ = -*1*, Ψ_3_ = *0.25*, *σ*_1_ = *2*, *σ*_2_ = *1*, *α* = *3*, *β* = *2*, Υ = *3*.

**Fig 12:**
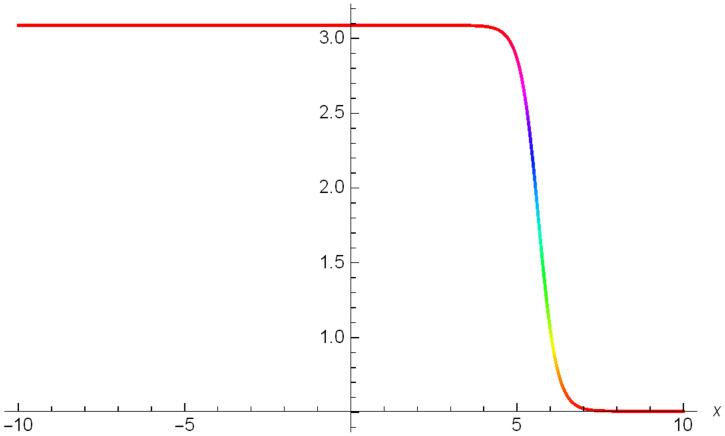
Solitary wave structure for the generalized Zakharov system (Case-II) in 2D visualization of ${|Y\left(x,t\right)}_{12}^{II}|$: Ψ_1_ = *1*, Ψ_2_ = -*1*, Ψ_3_ = *0.25*, *σ*_1_ = *2*, *σ*_2_ = *1*, *α* = *3*, *β* = *2*, Υ = *3*.

Similarly, Figs 10–12 demonstrate a solitary wave structure $|Y_{12}^{II}\left(x,t\right)|$ established in Case II for Ψ_1_ = *1*, Ψ_2_ = -*1*, Ψ_3_ = *0.25*, *σ*_1_ = *2*, *σ*_2_ = *1*, *α* = *3*, *β* = *2*, Υ = *3*. in 3D, Cantor and 2D formats respectively.

**Fig 13:**
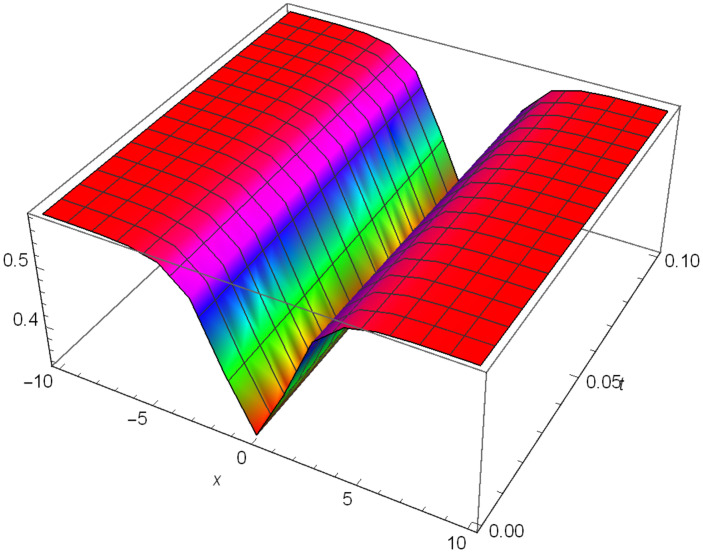
Dark wave structure for the generalized Zakharov system (Case-III Type I) in 3D visualization of ${|Y\left(x,t\right)}_{1}^{III}|$: Υ_1_ = *1*, Υ_2_ = *2*, Υ_3_ = *3*, *σ*_1_ = *1*, *σ*_2_ = *1*, *α* = *1*, *β* = *2*, Υ = -*1*.

**Fig 14:**
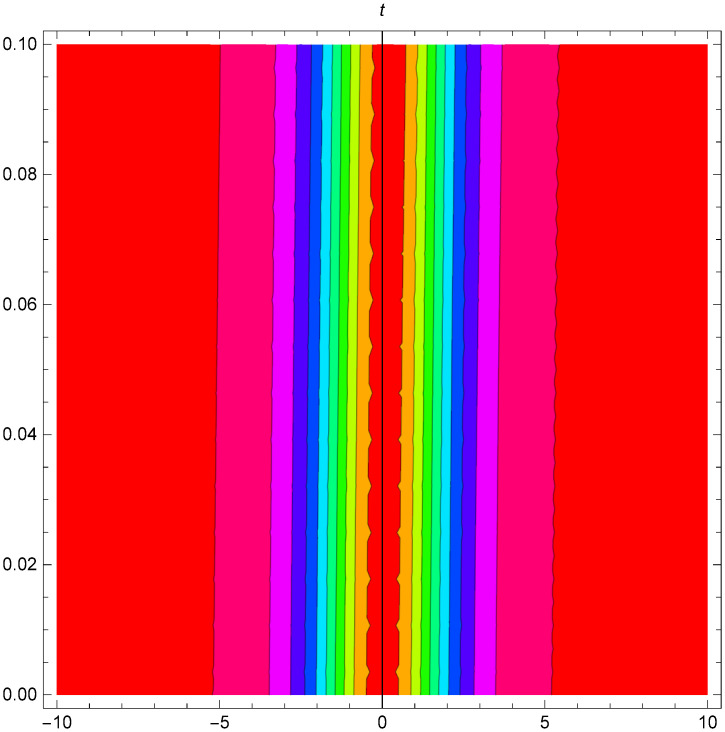
Dark wave structure for the generalized Zakharov system (Case-III Type I) in Cantor shape of ${|Y\left(x,t\right)}_{1}^{III}|$: Υ_1_ = *1*, Υ_2_ = *2*, Υ_3_ = *3*, *σ*_1_ = *1*, *σ*_2_ = *1*, *α* = *1*, *β* = *2*, Υ = -*1*.

**Fig 15:**
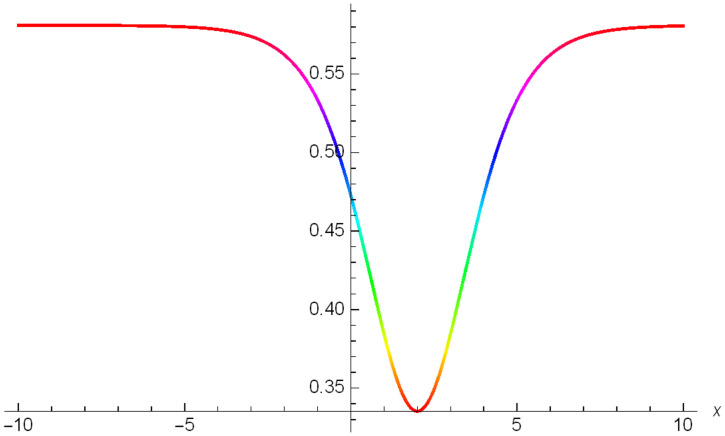
Dark wave structure for the generalized Zakharov system (Case-III Type I) in 2D visualization of ${|Y\left(x,t\right)}_{1}^{III}|$: Υ_1_ = *1*, Υ_2_ = *2*, Υ_3_ = *3*, *σ*_1_ = *1*, *σ*_2_ = *1*, *α* = *1*, *β* = *2*, Υ = -*1*.

While Figs 13–15 display a dark wave structure $|Y_{1}^{III}\left(x,t\right)|$ established in Case III (Type I) for Υ_1_ = *1*, Υ_2_ = *2*, Υ_3_ = *3*, *σ*_1_ = *1*, *σ*_2_ = *1*, *α* = *1*, *β* = *2*, Υ = -*1* in 3D, Cantor and 2D formats respectively.

**Fig 16:**
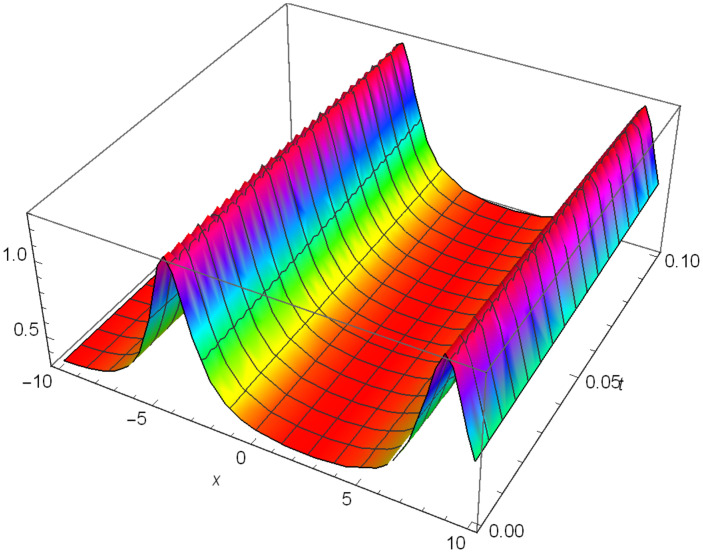
Double bell shaped wave structure for the generalized Zakharov system (Case-III Type VII) in 3D visualization of ${|Y\left(x,t\right)}_{7}^{III}|$: Υ_1_ = *0.5*, Υ_2_ = *1*, Υ_3_ = *2*, *σ*_1_ = *2.5*, *σ*_2_ = *0.5*, *α* = *1.25*, *β* = *2*, Υ = *0.25*.

**Fig 17:**
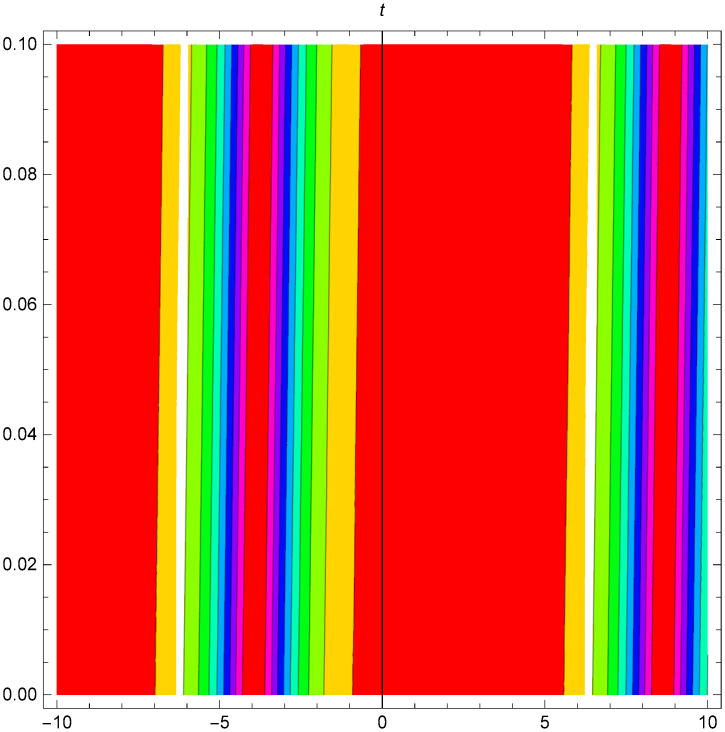
Double bell shaped wave structure for the generalized Zakharov system (Case-III Type VII) in Cantor shape of ${|Y\left(x,t\right)}_{7}^{III}|$: Υ_1_ = *0.5*, Υ_2_ = *1*, Υ_3_ = *2*, *σ*_1_ = *2.5*, *σ*_2_ = *0.5*, *α* = *1.25*, *β* = *2*, Υ = *0.25*.

**Fig 18:**
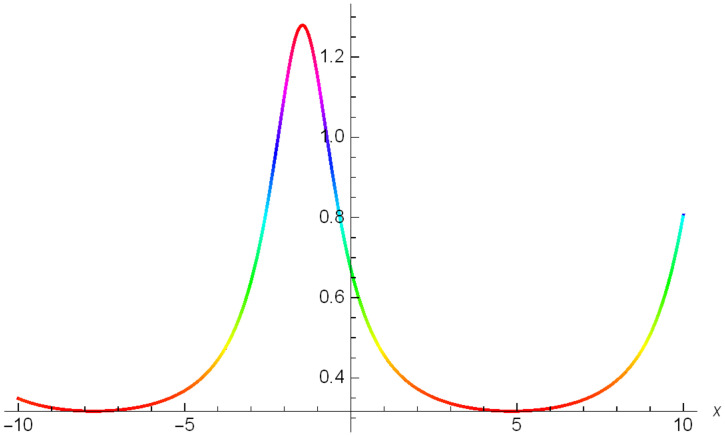
Double bell shaped wave structure for the generalized Zakharov system (Case-III Type VII) in 2D visualization of ${|Y\left(x,t\right)}_{7}^{III}|$: Υ_1_ = *0.5*, Υ_2_ = *1*, Υ_3_ = *2*, *σ*_1_ = *2.5*, *σ*_2_ = *0.5*, *α* = *1.25*, *β* = *2*, Υ = *0.25*.

Whereas Figs 16–18 present a double bell shaped wave structure $|Y_{7}^{III}\left(x,t\right)|$ established in Case III (Type VII) for Υ_1_ = *0.5*, Υ_2_ = *1*, Υ_3_ = *2*, *σ*_1_ = *2.5*, *σ*_2_ = *0.5*, *α* = *1.25*, *β* = *2*, Υ = *0.25* in 3D, Cantor and 2D formats respectively.

**Fig 19:**
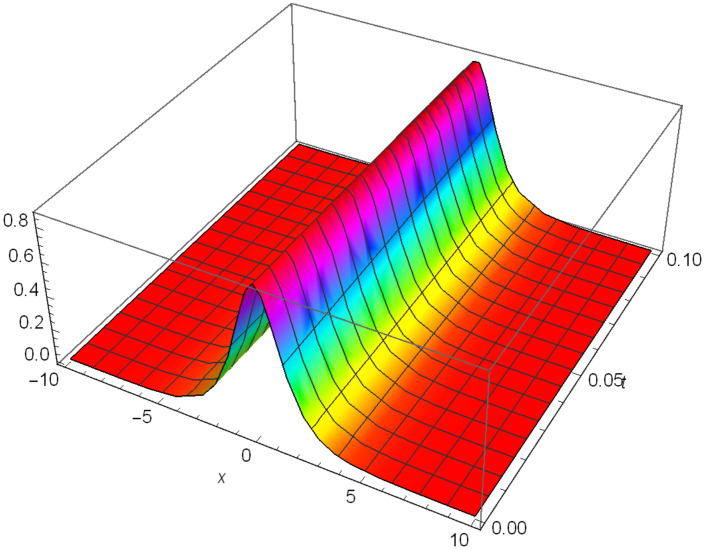
Bright bell shaped wave structure for the generalized Zakharov system (Case-IV Type II for *m* → 1) in 3D visualization of ${|Y\left(x,t\right)}_{2}^{IV}|$: *σ*_1_ = *1*, *σ*_2_ = *1*, *α* = *1*, *β* = *2*, Υ = -*1*.

**Fig 20:**
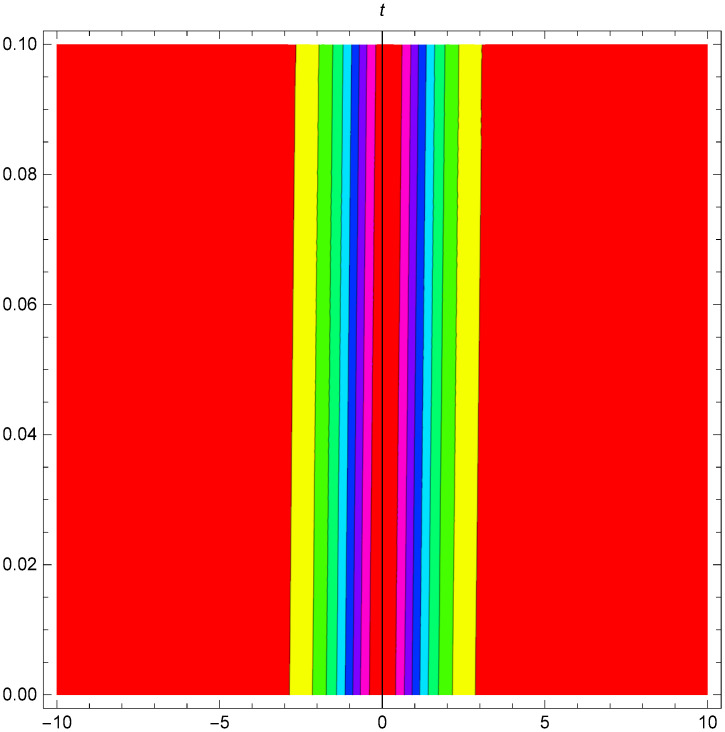
Bright bell shaped wave structure for the generalized Zakharov system (Case-IV Type II for *m* → 1) in Cantor shape of ${|Y\left(x,t\right)}_{2}^{IV}|$: *σ*_1_ = *1*, *σ*_2_ = *1*, *α* = *1*, *β* = *2*, Υ = -*1*.

**Fig 21:**
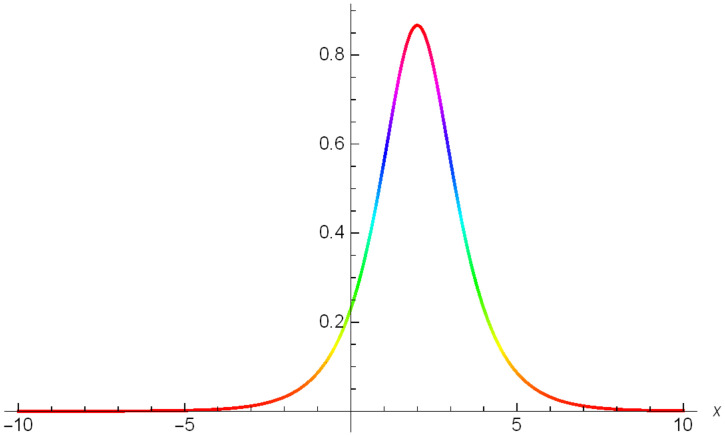
Bright bell shaped wave structure for the generalized Zakharov system (Case-IV Type II for *m* → 1) in 2D visualization of ${|Y\left(x,t\right)}_{2}^{IV}|$: *σ*_1_ = *1*, *σ*_2_ = *1*, *α* = *1*, *β* = *2*, Υ = -*1*.

Additionally, Figs 19–21 demonstrate a bell shaped bright wave structure $|Y_{2}^{IV}\left(x,t\right)|$ developed in Case IV for *σ*_1_ = *1*, *σ*_2_ = *1*, *α* = *1*, *β* = *2*, Υ = -*1* in 3D, Cantor and 2D formats respectively.

**Fig 22:**
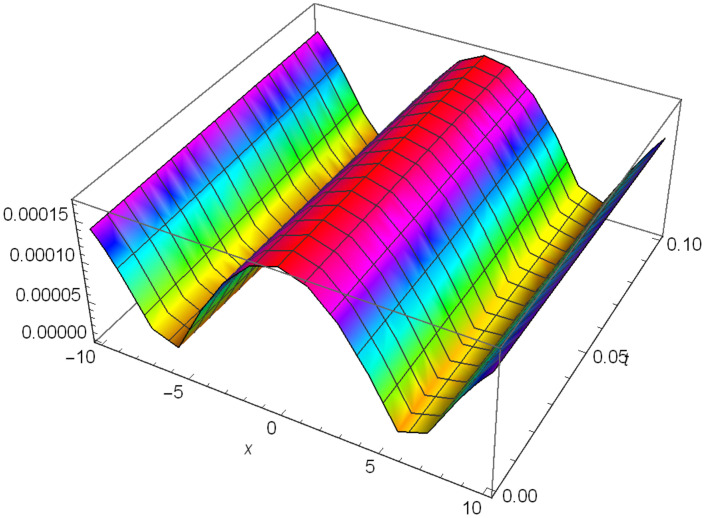
Periodic bright shaped wave structure for the generalized Zakharov system (Case-IV Type II for *m* → 0) in 3D visualization of ${|Y\left(x,t\right)}_{2}^{IV}|$: *σ*_1_ = *2*, *σ*_2_ = *3*, *α* = *1.5*, *β* = *2*, Υ = -*0.25*.

**Fig 23:**
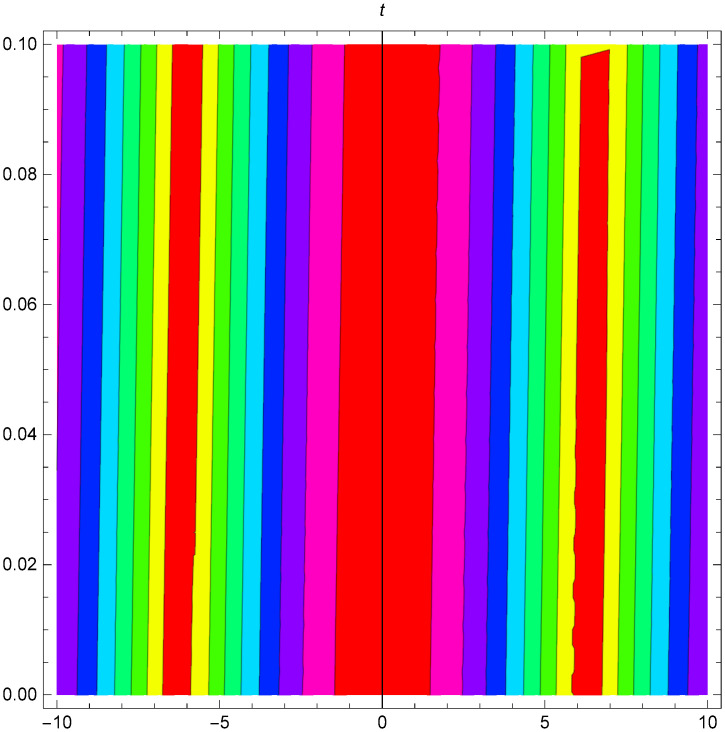
Periodic bright shaped wave structure for the generalized Zakharov system (Case-IV Type II for *m* → 0) in Cantor shape of ${|Y\left(x,t\right)}_{2}^{IV}|$: *σ*_1_ = *2*, *σ*_2_ = *3*, *α* = *1.5*, *β* = *2*, Υ = -*0.25*.

**Fig 24:**
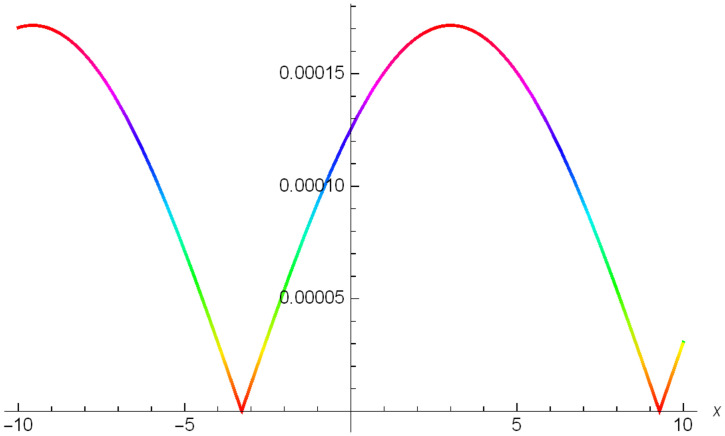
Periodic bright shaped wave structure for the generalized Zakharov system (Case-IV Type II for *m* → 0) in 2D visualization of ${|Y\left(x,t\right)}_{2}^{IV}|$: *σ*_1_ = *2*, *σ*_2_ = *3*, *α* = *1.5*, *β* = *2*, Υ = -*0.25*.

While Figs 22–24 present a periodic wave structure $|Y_{2}^{IV}\left(x,t\right)|$ developed in Case IV for *σ*_1_ = *2*, *σ*_2_ = *3*, *α* = *1.5*, *β* = *2*, Υ = -*0.25* in 3D, Cantor and 2D formats respectively.

## 4 Sensitivity analysis

The Galilean transformation turns the ODE 6 into a first-order dynamical system of the form:

$$\begin{array}{c} \left\{ \begin{array}{c} \begin{array}{cc} & \frac{dU}{d\xi }=P,\\ & \frac{dP}{d\xi }=-\theta_{1}U^{3}-\theta_{2}U, \end{array} \end{array}\right. \end{array}$$ (14)

where

$$\begin{array}{c} \theta_{1}=\frac{\delta_{1}}{\Upsilon^{2}},\;\;\theta_{2}=\frac{\delta_{2}}{\Upsilon^{2}}. \end{array}$$

**Fig 25:**
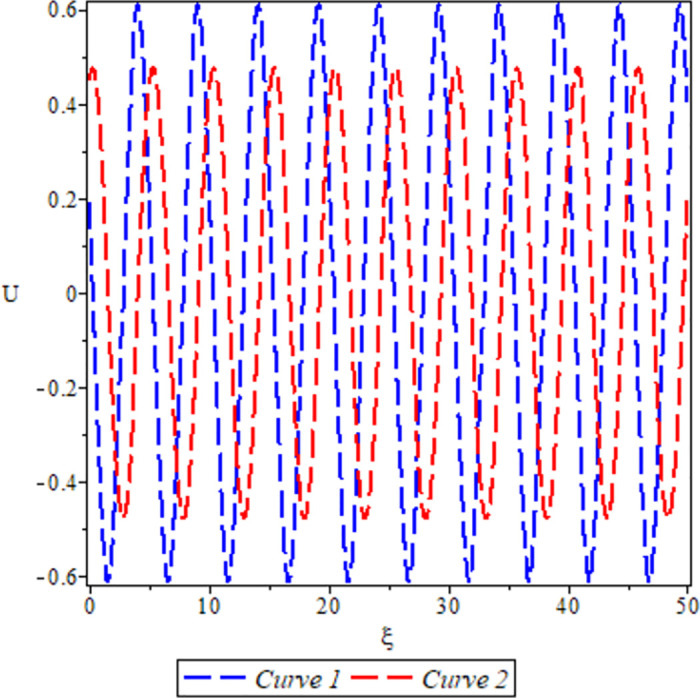
For *θ*_1_ < 0 and *θ*_2_ > 0.

**In** Fig 25, the sensitivity analysis of the dynamical system (14) is presented for conditions where either *θ*_1_ < 0, *θ*_2_ > 0 under *a* = 1, *σ*_1_ = 1, *β* = −1, *c* = 1, Υ = 1. The depiction highlights highly nonlinear periodic waves for two specific initial conditions: (*U*, *P*) = (0.44, 0.19) represented by the blue curve, and (*U*, *P*) = (0.45, 0.19) represented by the red curve. It is noteworthy that even a slight disparity in initial conditions significantly influences the solutions, underscoring the practical importance of phase portraits.

**Fig 26:**
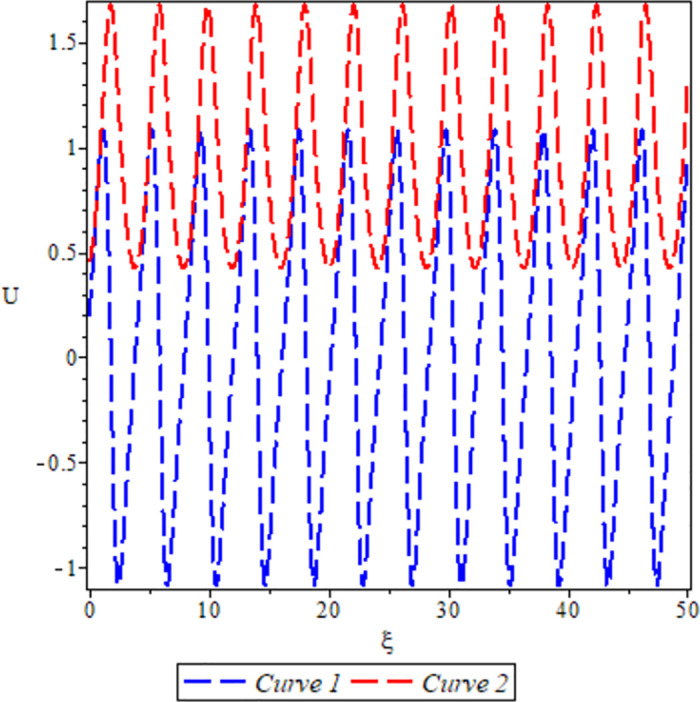
For *θ*_1_ > 0 and *θ*_2_ < 0.

**In** Fig 26, the sensitivity analysis of the dynamical system (14) is presented for conditions where either *θ*_1_ > 0, *θ*_2_ < 0 under *a* = 1, *σ*_1_ = −1, *β* = −1, *c* = −1, Υ = 1. The depiction highlights highly nonlinear periodic waves for two specific initial conditions: (*U*, *P*) = (0.44, 0.19) represented by the blue curve, and (*U*, *P*) = (0.45, 0.19) represented by the red curve. It is important to mention that even a slight disparity in initial conditions significantly influences the solutions, underscoring the practical importance of phase portraits.

**Fig 27:**
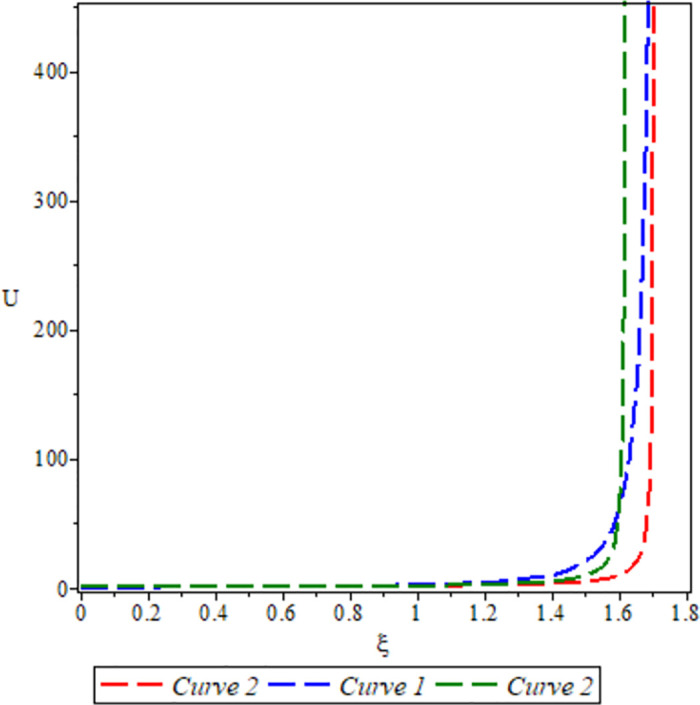
For *θ*_1_ < 0 and *θ*_2_ < 0.

**In** Fig 27, the sensitivity analysis of the dynamical system (14) is presented for conditions where either *θ*_1_ < 0 or *θ*_2_ < 0 under *a* = 1, *σ*_1_ = 0.5, *β* = −1, *c* = −1, Υ = 1. The depiction highlights highly nonlinear periodic waves for three specific initial conditions: (*U*, *P*) = (0.40, 0.15) represented by the blue curve, (*U*, *P*) = (0.42, 0.15) represented by the red curve, and (*U*, *P*) = (0.48, 0.15) represented by the green curve.

**Fig 28:**
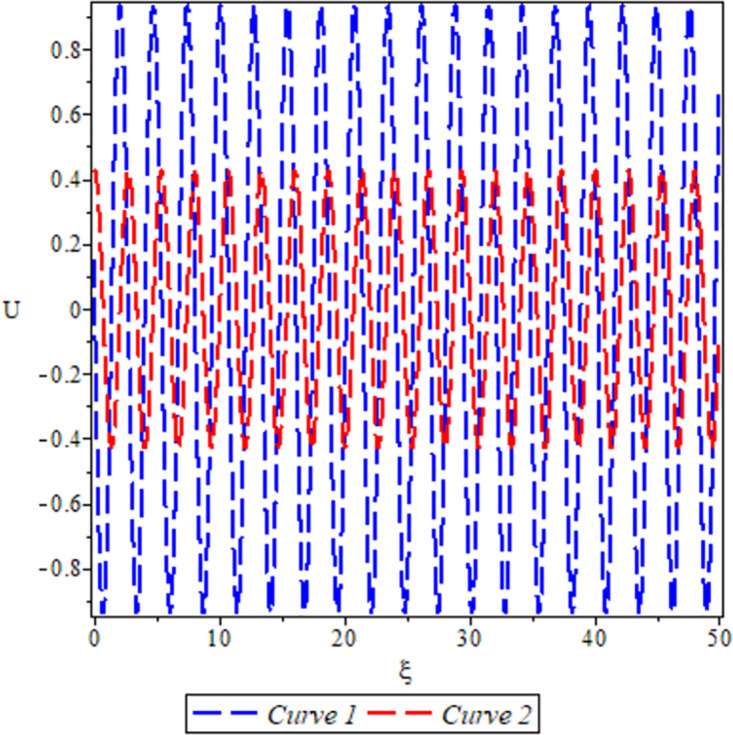
For *θ*_1_ > 0 and *θ*_2_ > 0.

**In** Fig 28, the sensitivity analysis of the dynamical system (14) is presented for conditions where either *θ*_1_ > 0, *θ*_2_ > 0 under *a* = 0, *σ*_1_ = −1, *β* = −1, *c* = 2, Υ = 1. The depiction highlights highly nonlinear periodic waves for two specific initial conditions: (*U*, *P*) = (0.13, 0.03) represented by the red curve, and (*U*, *P*) = (0.11, 0.02) represented by the green curve.

## 5 Quasi-periodic chaotic patterns

In this section, the perturbation component is introduced to the planar dynamical system (14). It is possible to formulate the planar dynamical system with the additional disturbance term as follows [26, 27]:

$$\begin{array}{c} \left\{ \begin{array}{c} \begin{array}{cc} & \frac{dU}{d\xi }=P,\\ & \frac{dP}{d\xi }=-\theta_{1}U^{3}-\theta_{2}U+\eta_{0}\;\text{cos}\left(\eta_{1}\xi \right), \end{array} \end{array}\right. \end{array}$$ (15)

where

$$\begin{array}{c} \theta_{1}=\frac{\delta_{1}}{\Upsilon^{2}},\;\;\theta_{2}=\frac{\delta_{2}}{\Upsilon^{2}}. \end{array}$$

In the above considered perturbed system (15), the effect part has two components, i.e. *η*_0_ and *η*_1_. While these parameters demonstrate both the magnitude and frequency of an external force acting on a dynamical system, respectively. Here, the effect of the frequency term on the considered model will be investigated. To do that we will keep the physical parameters of the model at their fixed values while we investigate the impact of perturbation’s amplitude and period.

**Fig 29:**
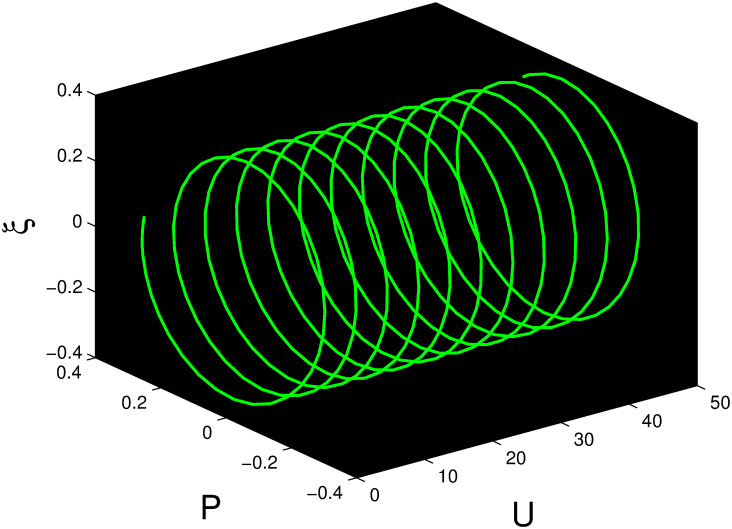
Three-dimensional phase portrait for nonlinear dynamical system (15) for *θ*_1_ < 0 and *θ*_2_ > 0 or *θ*_1_ > 0 and *θ*_2_ > 0 or *θ*_1_ < 0 and *θ*_2_ < 0.

**Fig 30:**
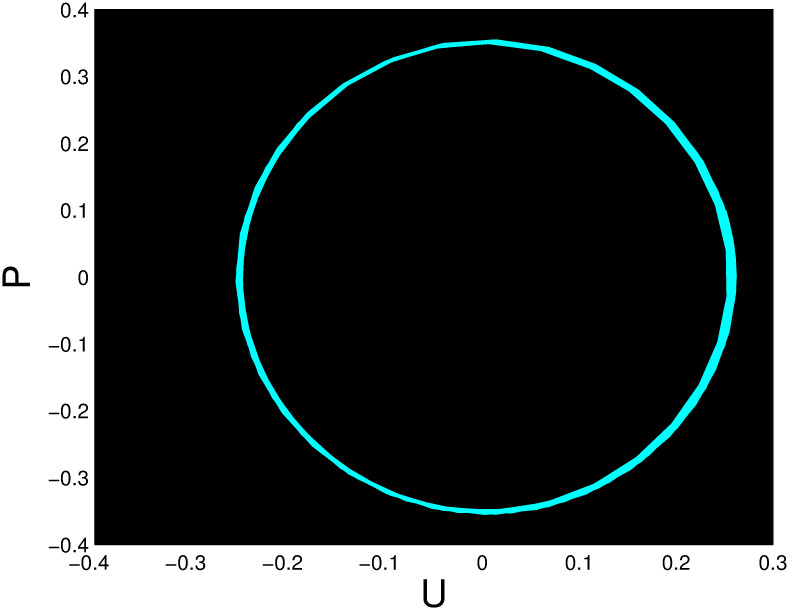
Two-dimensional phase portrait for nonlinear dynamical system (15) for *θ*_1_ < 0 and *θ*_2_ > 0 or *θ*_1_ > 0 and *θ*_2_ > 0 or *θ*_1_ < 0 and *θ*_2_ < 0.

**Fig 31:**
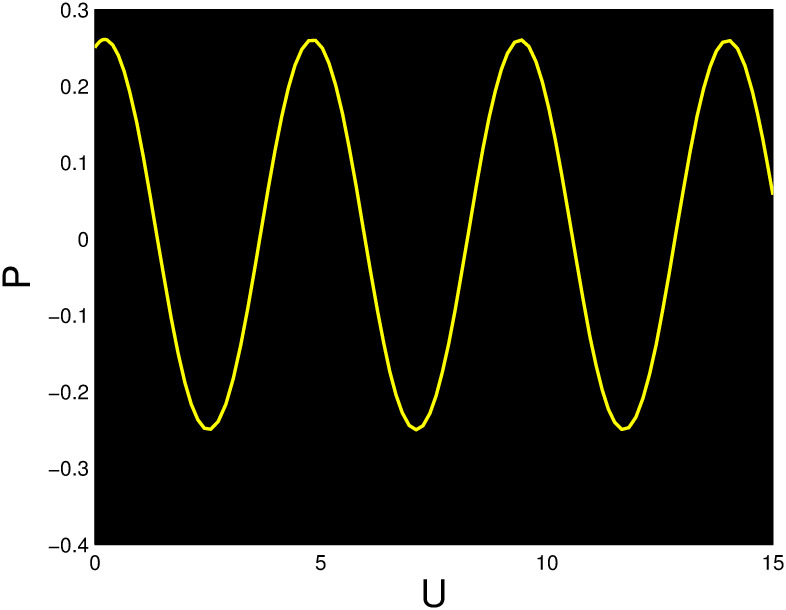
Time series when *t* = 15 for nonlinear dynamical system (15) for *θ*_1_ < 0 and *θ*_2_ > 0 or *θ*_1_ > 0 and *θ*_2_ > 0 or *θ*_1_ < 0 and *θ*_2_ < 0.

**Fig 32:**
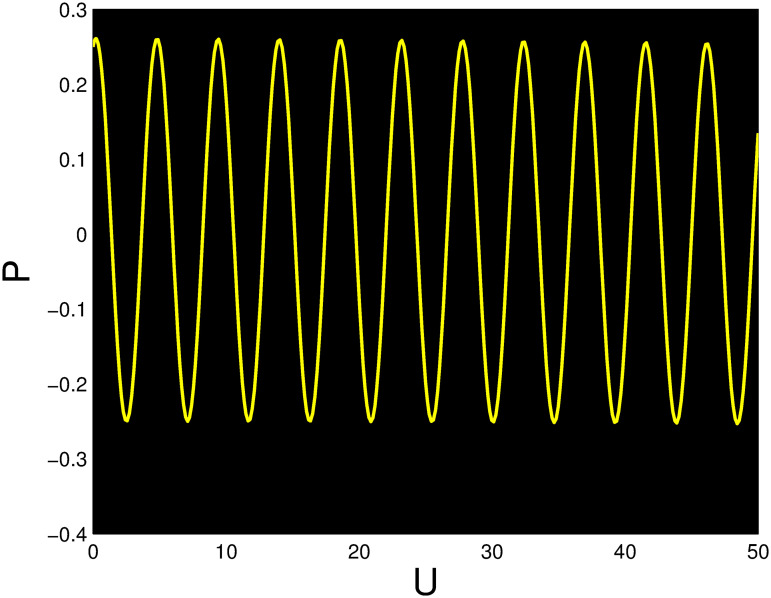
Time series when *t* = 50 for nonlinear dynamical system (15) for *θ*_1_ < 0 and *θ*_2_ > 0 or *θ*_1_ > 0 and *θ*_2_ > 0 or *θ*_1_ < 0 and *θ*_2_ < 0.

**Fig 33:**
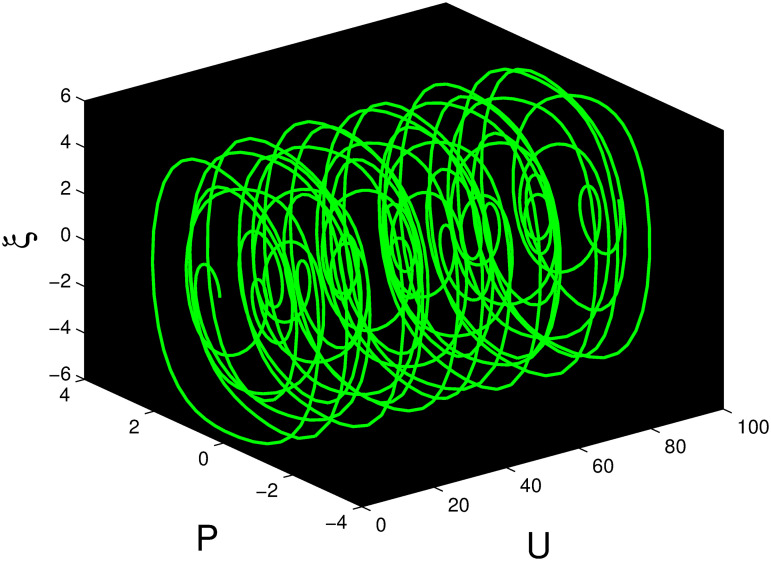
Three-dimensional phase portrait for nonlinear dynamical system (15) for *θ*_1_ > 0 and *θ*_2_ < 0.

**Fig 34:**
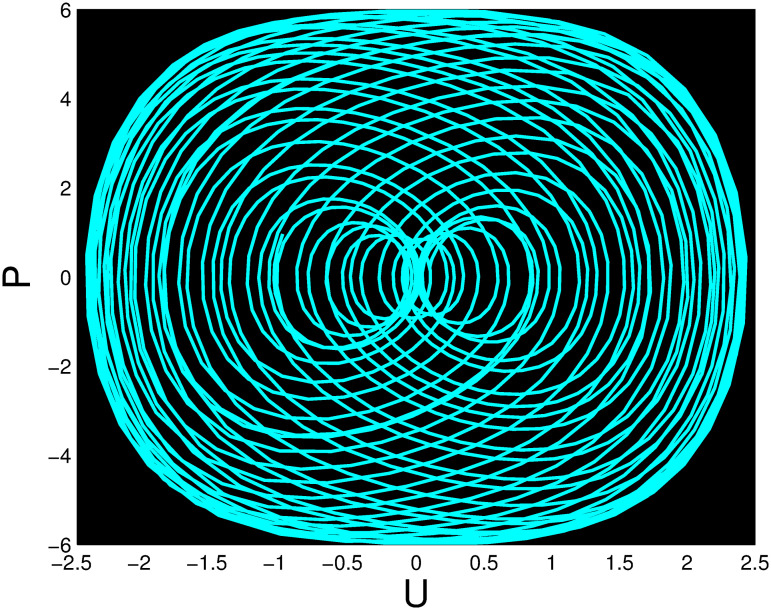
Two-dimensional phase portrait for nonlinear dynamical system (15) for *θ*_1_ > 0 and *θ*_2_ < 0.

**Fig 35:**
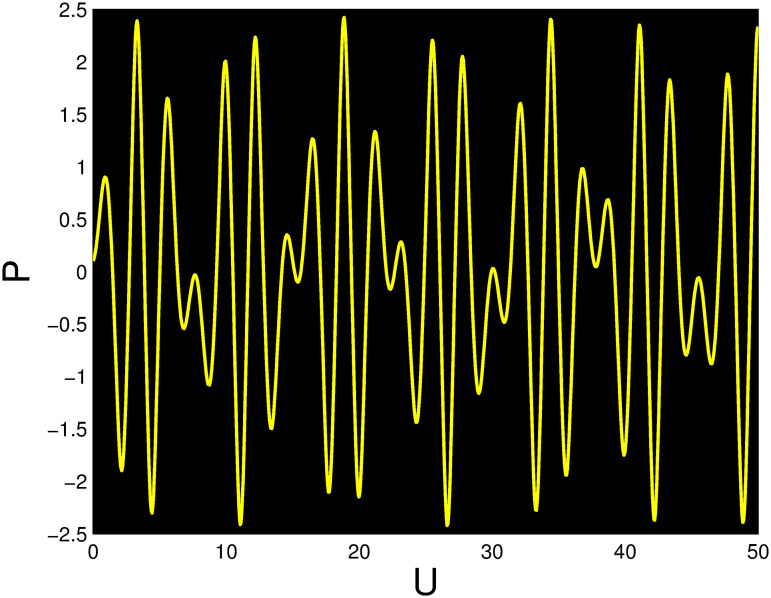
Time series when *t* = 15 for nonlinear dynamical system (15) for *θ*_1_ > 0 and *θ*_2_ < 0.

**Fig 36:**
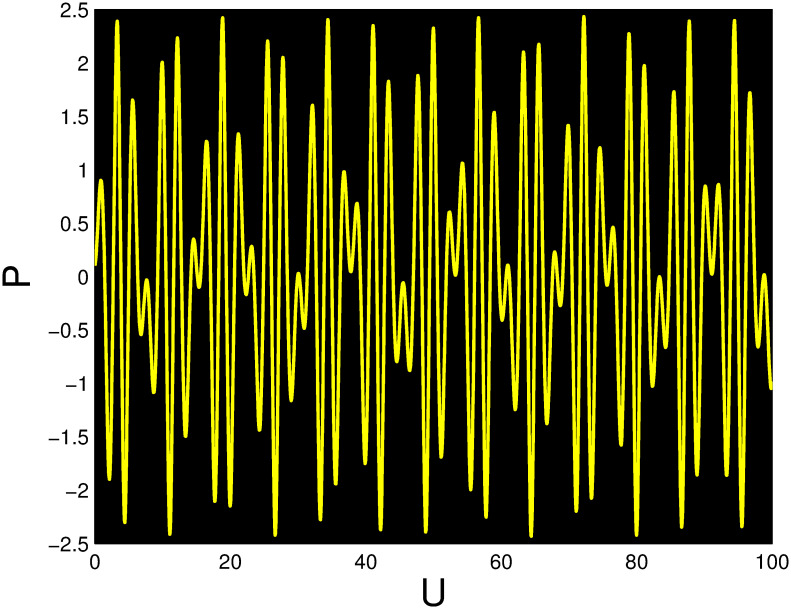
Time series when *t* = 50 for nonlinear dynamical system (15) for *θ*_1_ > 0 and *θ*_2_ < 0.

A time series, a two-dimensional phase portrait, and a three-dimensional phase portrait graph are shown for *η*_0_ = 0.01 and *η*_1_ = 0.03 with a starting condition of (*U*, *P*) = (0.25, 0.10), using the unique values of parameters, in Figs 29–32 respectively. It has been observed that the disturbed dynamical system (15) behaves periodically. The figures of the phase portraits in 2D and 3D, a time series from the *η*_0_ = 4, *η*_1_ = 2 case along with starting values of (*U*, *P*) = (0.10, 0.20) are illustrated in Figs 33–36 respectively.

## 6 Multistability

This study examines the multistability of an item with the disrupted term (15). Multistability describes the state where different solutions occur in a system of dynamics with different starting conditions.

**Fig 37:**
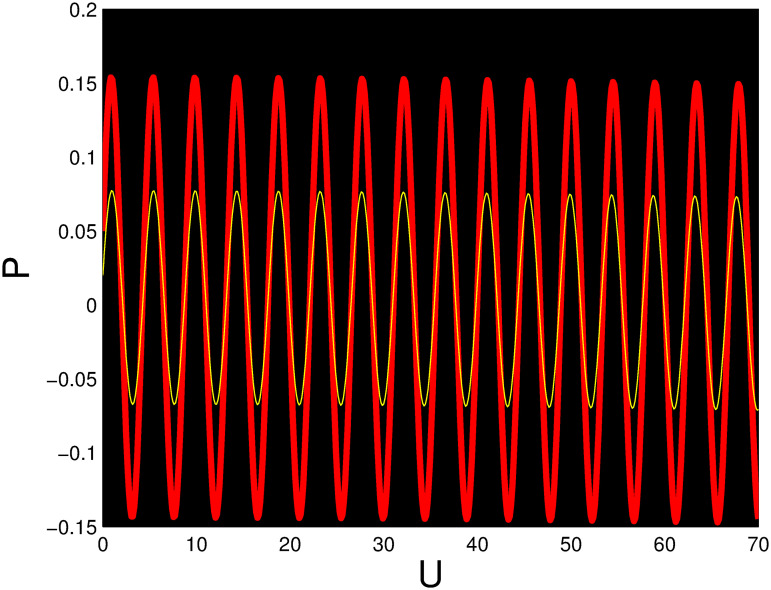
Time series graph for the multistability of the nonlinear dynamical system (15) with *η*_0_ = 0.01 and *η*_1_ = 0.02.

**Fig 38:**
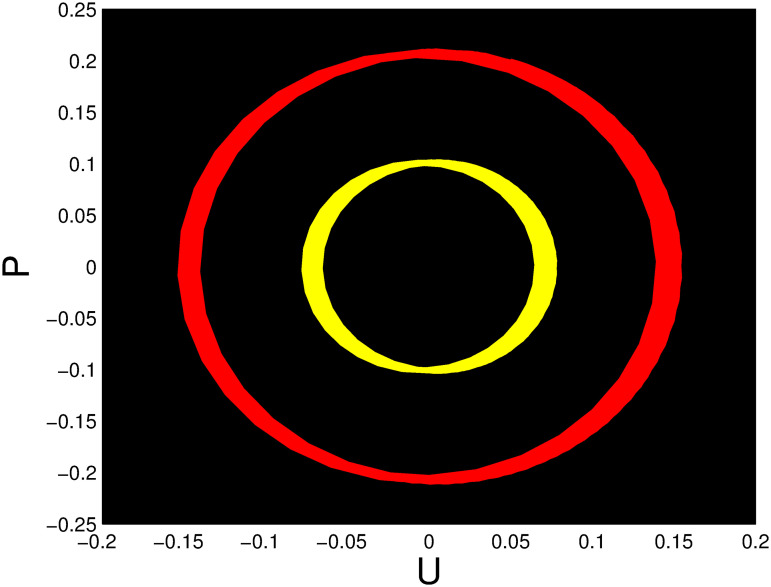
2D phase portrait of the multistability of the nonlinear dynamical system (15) with *η*_0_ = 0.01 and *η*_1_ = 0.02.

**Fig 39:**
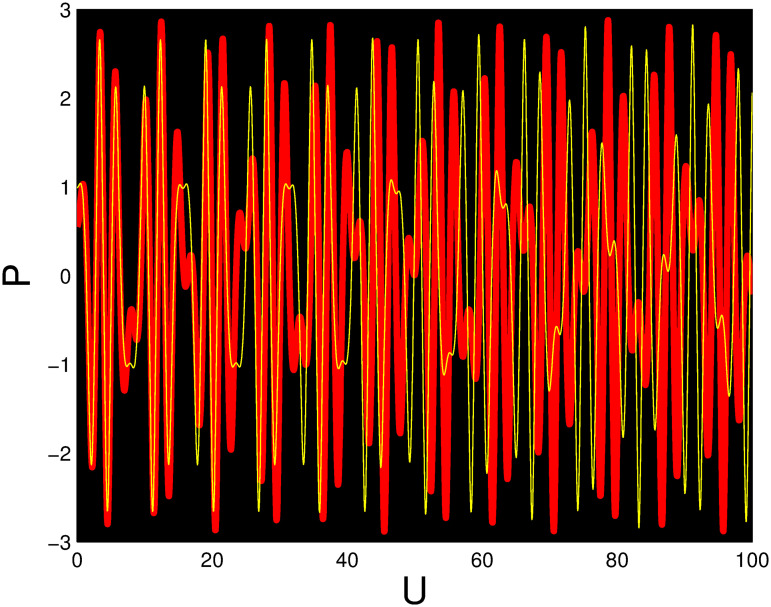
Time series graph for the multistability of the nonlinear dynamical system (15) with *η*_0_ = 4 and *η*_1_ = 2.

**Fig 40:**
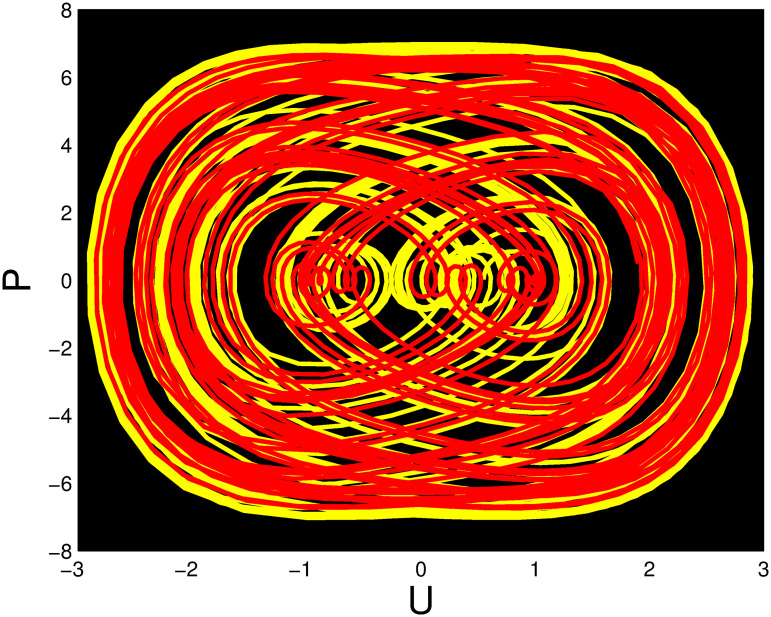
2D phase portrait of the multistability of the nonlinear dynamical system (15) with *η*_0_ = 4 and *η*_1_ = 2.

The red and yellow phase portraits has been displayed in Figs 37 and 38 correspond to *η*_0_ = 0.01 and *η*_1_ = 0.02 with initial conditions (*U*, *P*) = (0.05, 0.20) and (*U*, *P*) = (0.02, 0.10), respectively. It is observed that the system has a non-periodic behaviour when these initial conditions are selected. Figs 39 and 40 show phase portraits in red and green for *η*_0_ = 4 and *η*_1_ = 2 with initial conditions (*U*, *P*) = (0.55, 0.02) and (*U*, *P*) = (099, 0.02). The system displays periodic and quasi-periodic behavior for these initial conditions.

## 7 Lyapunov characteristic exponent

**Fig 41:**
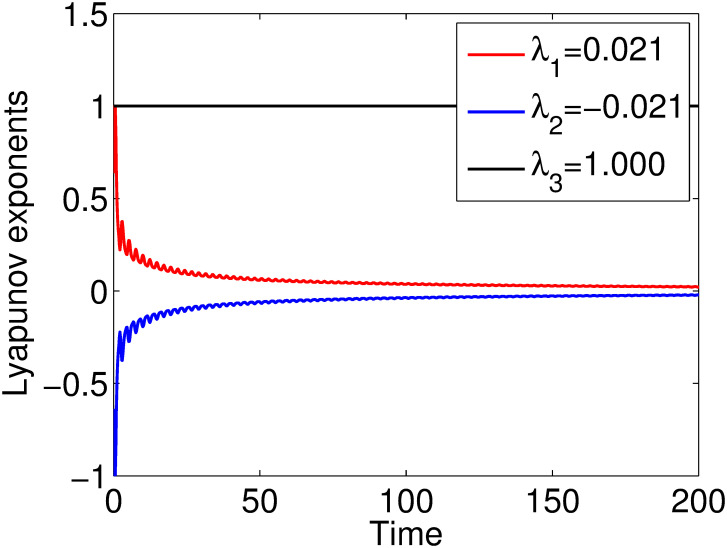
Dynamics of Lyapunov exponents.

Lyapunov characteristic exponent determines the diversion or convergence of neighboring trajectories with the same dynamics. Named after the Russian scientist A. Lyapunov, it indicates positive values for chaotic dynamics, negative values are for stability and zero value means marginal stability. It is undoubtedly a primary means in many fields of physical sciences and engineering. The maximum positive Lyapunov exponent is Υ = +0.021 which can be seen in Fig 41, indicating the presence of chaos.

The future development of Lyapunov indicators in all advanced fields seems to be promising. These exponents are empirically analyzed by researchers for an overall perception of the stability and adaptability of biological systems, potentially guiding the development of medicine and biotechnology. Incorporation of the Lyapunov exponent can lead to the improvement of the predictive ability of machines using AI or machine learning methods and open new perspectives in self-driving vehicles and intelligent systems. Additionally, in this investigation of Lyapunov exponents in quantum systems, innovations in quantum computing, and quantum communication technologies may be invented.

**Fig 42:**
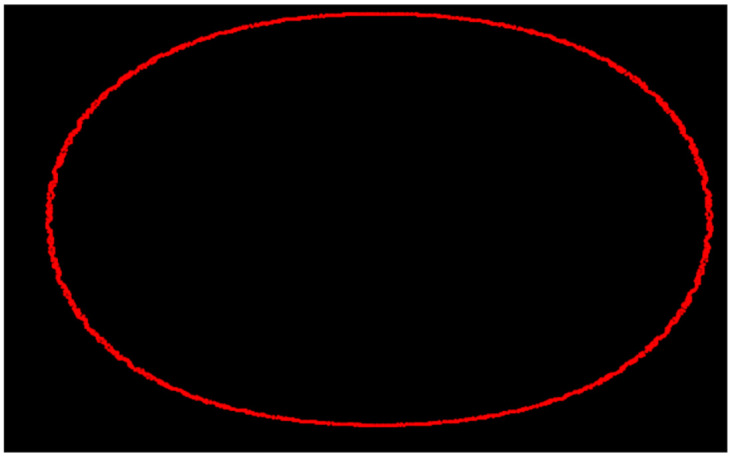
Poincaré map of the nonlinear dynamical system (15) for *θ*_1_ < 0 and *θ*_2_ > 0.

**Fig 43:**
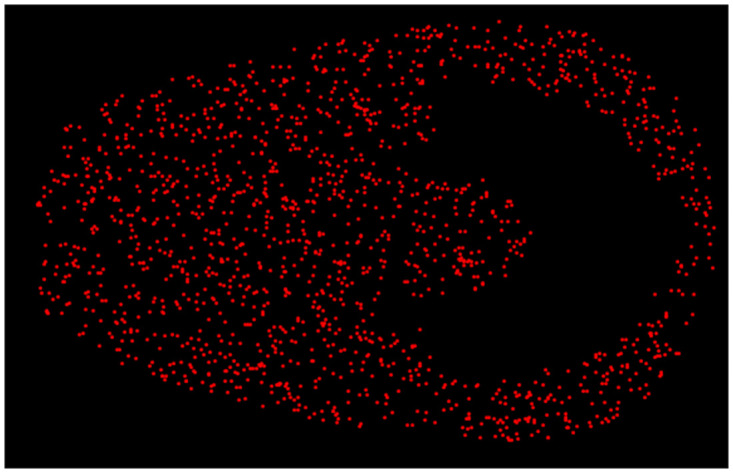
Poincaré map of the nonlinear dynamical system (15) for *θ*_1_ > 0 and *θ*_2_ < 0.

The Poincaré map is indispensable in the exploration of dynamical systems because of its capacity to simplify the dynamics of low-dimensional geometric figures. The Poincaré section of a trajectory on a plane shows the position of the fixed points, periodical orbits, or chaotic behavior. Moreover, the map provides stability analysis enabling researchers to identify the stability of system states and predict transition between different dynamical dynamic regimes. It is used in the determination of bifurcation analysis allowing us to know about the qualitative changes in the behavior of the system at the point of changing parameters. In essence, the Poincaré map is a vital instrument used for achieving in-depth knowledge and accurate prediction of the dynamical behavior of systems in many branches of science. Figs 42 and 43 show chaotic behavior using a Poincaré map at differemt conditions.

## 8 Conclusions

In this work, the extended Zakharov system that describes the propagation of ion and dispersive acoustic waves in plasma is studied. A highly oscillatory electric field’s fluctuating envelope is represented by the complex, dispersive field *Y*, whereas the change of the plasma ion density from its equilibrium state is represented by the real, non-dispersive field *Z*. We employed the extended Fan sub-equation method to unveil numerous novel traveling wave solutions within the framework of the governing complex system. Across a wide range of parameters, our research produced a broad collection of exact solutions, including explicit, periodic, and linked wave solutions. Three-dimensional graphical analyses were used to clarify the features of these solutions. In addition, we used ideas from planar dynamical theory to explore the model’s complex dynamics. We were able to understand the behaviour of the system by performing sensitivity analysis, investigating multistability, looking at quasi-periodic and chaotic patterns, and calculating the Lyapunov characteristic exponent. Different beginning circumstances were used to study sensitivity and multistability, and the system’s chaotic behaviour was examined by adding an external periodic force. For chaos identification, a number of methods were employed including time series plots, Lyapunov exponents, and 3D and 2D phase diagrams. Our results show that the system displays irregular behaviour, deviations from traditional modes, and chaotic dynamics over extended periods of time. These findings highlight how effectively the techniques deployed to address these kinds of higher-dimensional, complicated nonlinear dynamical models work in modern applied science and engineering fields.
